# Chromatin-Specific Regulation of Mammalian rDNA Transcription by Clustered TTF-I Binding Sites

**DOI:** 10.1371/journal.pgen.1003786

**Published:** 2013-09-12

**Authors:** Sarah D. Diermeier, Attila Németh, Michael Rehli, Ingrid Grummt, Gernot Längst

**Affiliations:** 1 Biochemistry Centre Regensburg (BCR), University of Regensburg, Regensburg, Germany; 2 Department of Hematology, University Hospital Regensburg, Regensburg, Germany; 3 Molecular Biology of the Cell II, German Cancer Research Centre (DKFZ), Heidelberg, Germany; The University of North Carolina at Chapel Hill, United States of America

## Abstract

Enhancers and promoters often contain multiple binding sites for the same transcription factor, suggesting that homotypic clustering of binding sites may serve a role in transcription regulation. Here we show that clustering of binding sites for the transcription termination factor TTF-I downstream of the pre-rRNA coding region specifies transcription termination, increases the efficiency of transcription initiation and affects the three-dimensional structure of rRNA genes. On chromatin templates, but not on free rDNA, clustered binding sites promote cooperative binding of TTF-I, loading TTF-I to the downstream terminators before it binds to the rDNA promoter. Interaction of TTF-I with target sites upstream and downstream of the rDNA transcription unit connects these distal DNA elements by forming a chromatin loop between the rDNA promoter and the terminators. The results imply that clustered binding sites increase the binding affinity of transcription factors in chromatin, thus influencing the timing and strength of DNA-dependent processes.

## Author Summary

The sequence-specific binding of proteins to regulatory regions controls gene expression. Binding sites for transcription factors are rather short and present several million times in large genomes. However, only a small number of these binding sites are functionally important. How proteins can discriminate and select their functional regions is not clear, to date. Regulatory loci like gene promoters and enhancers commonly comprise multiple binding sites for either one factor or a combination of several DNA binding proteins, allowing efficient factor recruitment. We studied the cluster of TTF-I binding sites downstream of the rRNA gene and identified that cooperative binding to the multimeric termination sites in combination with low-affinity binding of TTF-I to individual sites upstream of the gene serves multiple regulatory functions. Packaging of the clustered sites into chromatin is a prerequisite for high-affinity binding, coordinated activation of transcription and the formation of a chromatin loop between the promoter and the terminator.

## Introduction

An intriguing question for understanding protein-DNA recognition is how low-abundant transcription factors recognize their target sites in genomic DNA [1], [2]. Empirical studies revealed that regulatory regions, such as enhancers and promoters, comprise modular units of a few hundred base pairs that harbour multiple binding sites for the same transcription factor. Such ‘homotypic clustering sites’ (HTCs) have been identified in 2% of the human genome, being enriched at promoters and enhancers [3]. HTCs have been shown to play a role in *Drosophila* development, regulating early patterning genes [4]–[6]. Genome-wide binding analyses in yeast have demonstrated that the occupancy of transcription factors is higher at clustered binding sites compared to single ones [7]. Studies in mammalian cells have shown that clustering of binding sites facilitate the cooperative binding of nuclear receptors to their target sites *in vivo*, suggesting that HCTs coordinate the recruitment of transcription initiation factors [8]–[10]. Alternatively, cooperative binding could arise through indirect effects, e.g. by changing the accessibility of neighbouring binding sites in chromatin [11].

To assess the functional relevance of homotypic clustering of transcription factor binding sites, we studied the 3′-terminal region of murine rRNA genes (rDNA), which contains ten binding sites (T_1_–T_10_) for the transcription termination factor TTF-I. Binding of TTF-I to the terminator elements is required to stop elongating RNA polymerase I (Pol I) and termination of pre-rRNA synthesis occurring predominantly at the first terminator T_1_ [12]–[15]. In addition to the downstream terminators, there is a single TTF-I binding site, termed T_0_, located 170 bp upstream of the transcription start site [16]. Binding of TTF-I to this site is required for efficient transcription initiation and for the recruitment of chromatin remodelling complexes that establish distinct epigenetic states of rRNA genes. The interaction of TTF-I with CSB (Cockayne Syndrome protein B), NoRC (Nucleolar Remodeling Complex), or NuRD (Nucleosome Remodeling and Deacetylation complex), respectively, has been shown to recruit histone modifying enzymes which lead to the establishment of a specific epigenetic signature that characterizes active, silent or poised rRNA genes [17]–[20].

TTF-I has been shown to oligomerize *in vitro* and to link two DNA fragments in *trans* [21]. These characteristics enable TTF-I bound to the upstream binding site T_0_ and the downstream terminators T_1_–T_10_ to loop out of the pre-rRNA coding region [22], [23]. Formation of a chromatin loop facilitates re-initiation and increases transcription initiation rates at the rRNA gene [22], [24]. TTF-I is a multifunctional protein that is not only essential for transcription termination, but also directs efficient rDNA transcription, mediates replication fork arrest [25], establishes specific epigenetic features and determines the topology of rDNA. The conservation of multiple TTF-I binding sites downstream of the pre-rRNA coding region raises the question whether homotypic clustering of terminator elements is functionally relevant. Here we demonstrate that HTCs serve a chromatin-specific function. Packaging into chromatin increases the binding affinity of TTF-I to clustered terminator elements, augments the efficiency of transcription termination, enhances transcription initiation, and changes the higher-order structure of rRNA genes. The homotypic clusters at the rRNA gene coordinate the timing of molecular events, coordinating transcription termination and initiation and the occurrence of higher-order chromatin domains, suggesting an important chromatin-dependent role for clustered binding sites in the genome.

## Results

### Multiple termination sites enhance the efficiency of transcription *in vitro*

**Figure 1:**
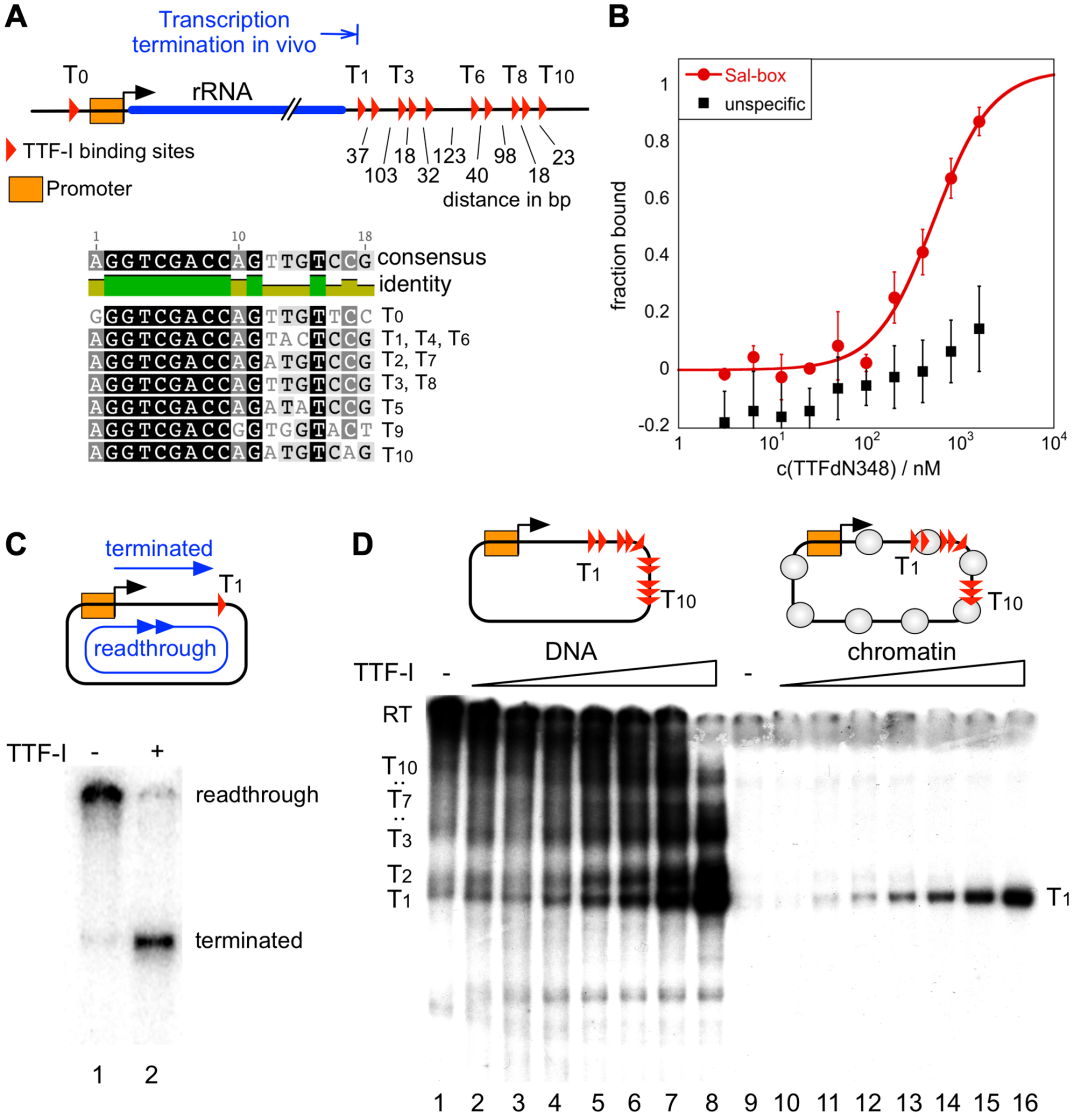
Chromatin-specific termination at the homotypic cluster of TTF-I. (A) Overview of the murine rRNA gene and the location of the TTF-I binding sites. A homotypic cluster of TTF-I sites is located in the terminator region. The distances between TTF-I binding sites, their orientation and the gene promoter are indicated. A comparison of the TTF-I binding sites T_0_ and the termination sites T_1_ to T_10_ is depicted. (B) Increasing amounts of TTF-IΔN348 were incubated with 50 nM of either a fluorescently labelled 30-mer oligonucleotide containing a Sal-box motif (T_2_) or a control oligonucleotide of the same length. Protein-DNA interactions are quantified by microscale thermophoresis. Curve fitting with a Hill coefficient of 1 resulted in a K_D_ of 500 nM+/−120 nM for the T_2_ sequence. (C) Transcription reaction using the circular rDNA minigene plasmid pMr-SB containing a single termination site, a partially purified nuclear extract lacking most of the nuclear TTF-I (DEAE280), performed in the presence or absence of recombinant TTF-I. The positions of the long read-through and the terminated transcripts are indicated. (D) Transcription on free DNA and chromatin, using the pMrWT-T DNA containing the promoter with the TTF-I binding site T_0_ and the full terminator with the 10 termination sites. DNA (lanes 1–8) and chromatin (lanes 9–16) were incubated with increasing concentrations of TTF-I as indicated and the DEAE280 extract. The position of the long, non-terminated read-through transcript (RT) and the terminated transcripts are indicated.

The rDNA terminators in human and mouse exhibit an overall similar structure, containing 10 to 11 TTF-I binding sites in close proximity (Fig. S1A). We focused on the murine rDNA terminator, which comprises 10 termination sites (T_1_–T_10_) spaced by 18–123 bp, preventing the accommodation of nucleosomes in between the TTF-I binding sites. The consensus sequence of these TTF-I binding sites share almost perfect sequence identity within the core motif GGTCGACCAG, while the surrounding nucleotides vary slightly (Fig. 1A). In electrophoretic mobility shift assays (EMSAs) recombinant TTF-I bound with comparable affinity to all terminators assayed (data not shown). The DNA binding affinity of TTF-I was quantified by microscale thermophoresis, recording changes of nucleoprotein complex mobility in a small temperature gradient [26]. By titrating a wide range of TTF-I∶DNA ratios, the binding constant of TTF-I to free Sal-box DNA was determined to be 0.5 µM (Fig. 1B), a relatively low DNA binding affinity which is one order of magnitude lower than the K_D_ of other transcription factors [27]–[29].

*In vitro* transcription assays on a circular minigene comprising the rDNA promoter fused to a single termination site (pMrSB) yielded long read-through transcripts in the absence of TTF-I. The addition of recombinant TTF-I led to the synthesis of terminated transcripts whose lengths correspond to the distance from the transcription start site to the terminator T_1_ (Fig. 1C). If the template contained all ten terminators (pMrT_1_-T_10_), both read-through transcripts and a heterogeneous population of transcripts randomly terminated at any of the TTF-I binding sites were synthesized due to sub-saturating TTF-I levels in the extract (Fig. 1D). In the presence of increasing concentrations of recombinant TTF-I the amount of transcripts stopped at terminator T_1_ progressively increased (Fig. 1D, lanes 1–8 and Fig. S2). Thus, TTF-I binds to all sites with similar affinity and randomly terminates transcription until at saturating concentrations TTF-I occupies all ten terminators.

A strikingly different result was obtained on rDNA templates assembled into chromatin with an extract from *Drosophila* embryos [30] (Figure S1B). Consistent with Pol I transcription on chromatin requiring binding of TTF-I to the promoter-proximal terminator T_0_ and ATP-dependent chromatin remodelling [31], [32], transcription was repressed in the absence of TTF-I (Fig. 1D, lane 9). The addition of TTF-I relieved transcriptional repression, yielding only a single RNA species of 686 nt. On chromatin templates, already lowest TTF-I concentrations terminated transcription specifically at T_1_ (Fig. 1D, lanes 10–16 and Fig. S2). The result suggests that transcription in chromatin is only initiated when the termination sites are set, meaning that the TTF-I binding site at the promoter is only bound after sequestering TTF-I at the terminator. The qualitative difference between transcription on free DNA and chromatin templates indicates that on chromatin templates TTF-I either binds preferentially to T_1_ or the overall binding affinity of TTF-I to all terminator sites is increased in chromatin.

### Clustered termination sites facilitate cooperative binding of TTF-I to chromatin

**Figure 2:**
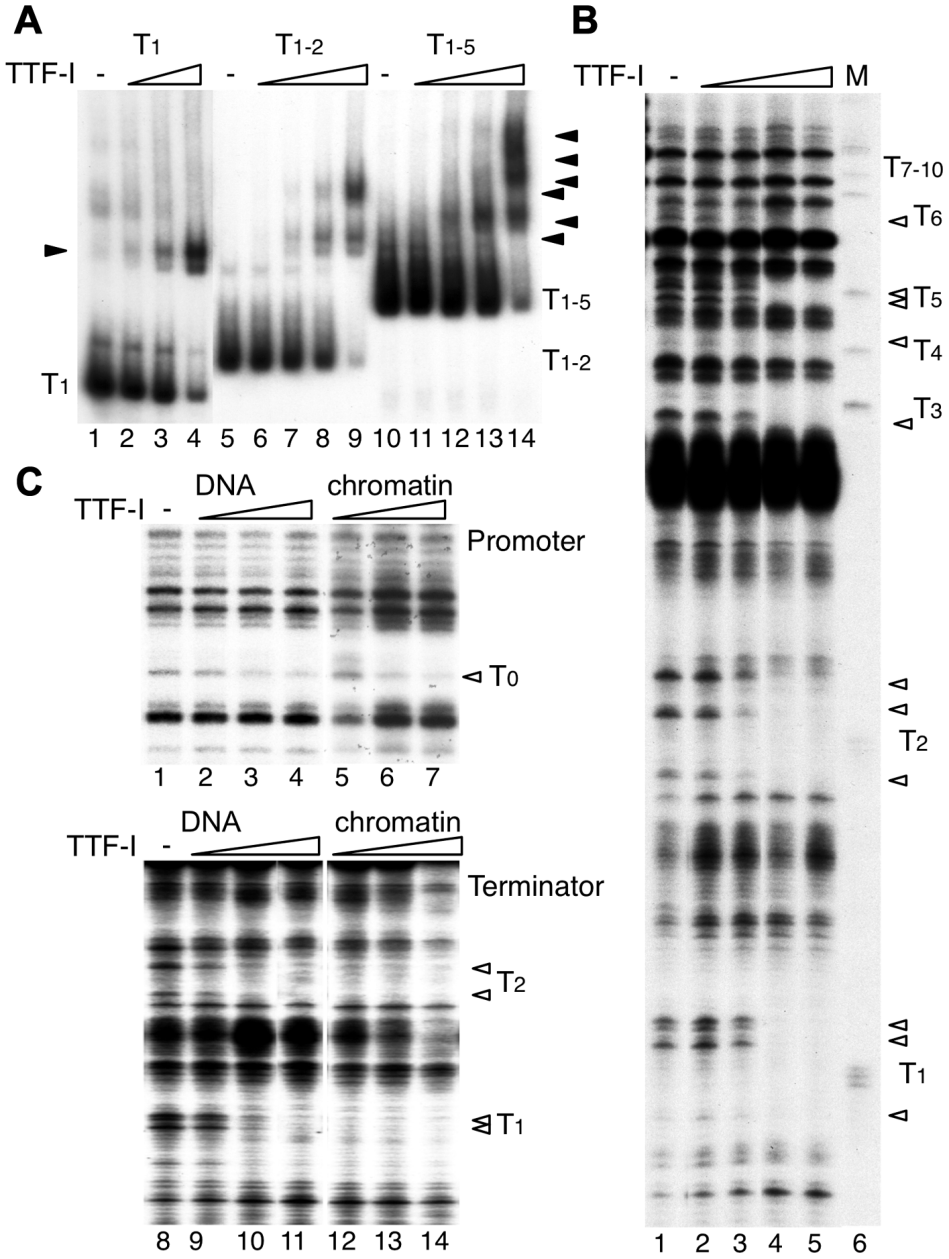
Multiple termination sites enable cooperative binding of TTF-I to chromatin. (A) Electrophoretic mobility shift assays (EMSA) were performed with a single TTF-I binding site (T_1_, lanes 1–4), two binding sites (T_1–2_, lanes 5–9) and an array of five binding sites (T_1–5_, lanes 10–14) and increasing concentrations of TTF-I as indicated. Nucleoprotein complexes are resolved on native polyacrylamide gels and detected by autoradiography. The positions of the free DNA molecules and the TTF-I-DNA complexes (triangles) are indicated. (B) Monitoring TTF-I binding to the chromatinized terminator by DNase I footprinting. The pMr-T plasmid containing the full terminator was reconstituted into chromatin with *Drosophila* embryo extract. Chromatin was incubated with increasing concentrations of TTF-I as indicated and partially digested with DNase I. Footprints were analysed by a primer extension reaction using a radioactively labelled oligonucleotide and resolving the DNA on 6% sequencing gels. The marker was generated by partial digestion of the plasmid with the restriction enzyme *Sal*I and analysed by the same primer extension reaction. The *Sal*I sites (T_1_ to T_10_) represent the TTF-I binding sites and the triangles indicate sites of DNase I protection. (C) Comparative footprinting of TTF-I binding to the promoter and terminator of free DNA and chromatin. Identical amounts of pMrWT-T were used as free DNA (lanes 1 to 4 and 8 to 11) or chromatin (lanes 5 to 7 and 12 to 14) and incubated with increasing amounts of TTF-I as indicated. DNA was partially digested with DNase I and the purified DNA was analysed by primer extension reactions, either using a radiolabelled oligonucleotide binding close to the promoter (lanes 1 to 7) or binding close to T_1_ in the terminator region (lanes 8 to 14). DNA was separated on 8% sequencing gels, dried and analysed after autoradiography. The TTF-I binding sites T_1_, T_2_ and T_0_ and the protected DNase I cutting sites (triangles) are indicated.

Next, we performed electrophoretic mobility shift assays (EMSAs) and DNase I footprinting experiments to compare TTF-I binding to free DNA and chromatin. Consistent with the transcription data on free DNA, EMSAs on terminator DNA fragments containing more than one TTF-I binding sites yielded heterogeneous nucleoprotein complexes, reflecting binding to each binding site with similar affinity (Fig. 2A). On chromatin templates, DNase I footprinting experiments demonstrate that TTF-I simultaneously bound to all terminator binding sites (Fig. 2B). Together with the transcription results on chromatin templates, this suggests that homotypic clustering of target sites increases the binding affinity of TTF-I to chromatin.

To compare the binding affinity of TTF-I to free DNA and reconstituted chromatin, we performed DNase I footprinting assays, monitoring DNase I cleavage sites by primer extension which allows simultaneous analysis of TTF-I occupancy at the promoter and terminator(s) (Fig. 2C). TTF-I binding can be observed by the disappearance of a DNase I sensitive site that is apparent within the TTF-I binding sites of free DNA and reconstituted chromatin (Fig. 2B and C). In agreement with the binding studies and the *in vitro* transcription experiments, TTF-I binds on free DNA to the promoter-proximal terminator T_0_ and the downstream terminators with similar affinity (Fig. 2C, compare lanes 2–4 and lanes 9–11). On chromatin templates, TTF-I binding to the upstream site T_0_ is comparable to its binding to free DNA (Fig. 2C, upper panel). However, on chromatin templates TTF-I binds with higher affinity to the clustered sites, fully occupying all terminator sites at low protein concentrations (Fig. 2C, lower panel). Significantly, TTF-I occupied the binding sites at the terminators prior to the promoter-proximal site (compare lanes 5–7 and 12–14), showing a specific role of chromatin and binding site clustering for increasing the binding affinity of TTF-I. The sequential binding of TTF-I, first to the terminators and then to the gene promoter in chromatin was also confirmed using a different method. Affinity purification of either TTF-I bound free DNA or chromatin revealed binding of TTF-I to the gene terminators reconstituted into chromatin already at concentrations one order of magnitude lower than with the gene promoter (Fig. S3). Like in the footprinting assay, this effect was not detectable using free DNA, where both TTF-I binding regions were occupied with similar affinity. Apparently, the clustered arrangement of binding sites increases the affinity of TTF-I, thus promoting the association of TTF-I with the downstream terminators T_1–10_ prior to the upstream site T_0_, a process that appears to be essential for both TTF-I dependent transcription activation and transcription termination.

### Clustered terminators act as a transcriptional enhancer *in vivo*

**Figure 3:**
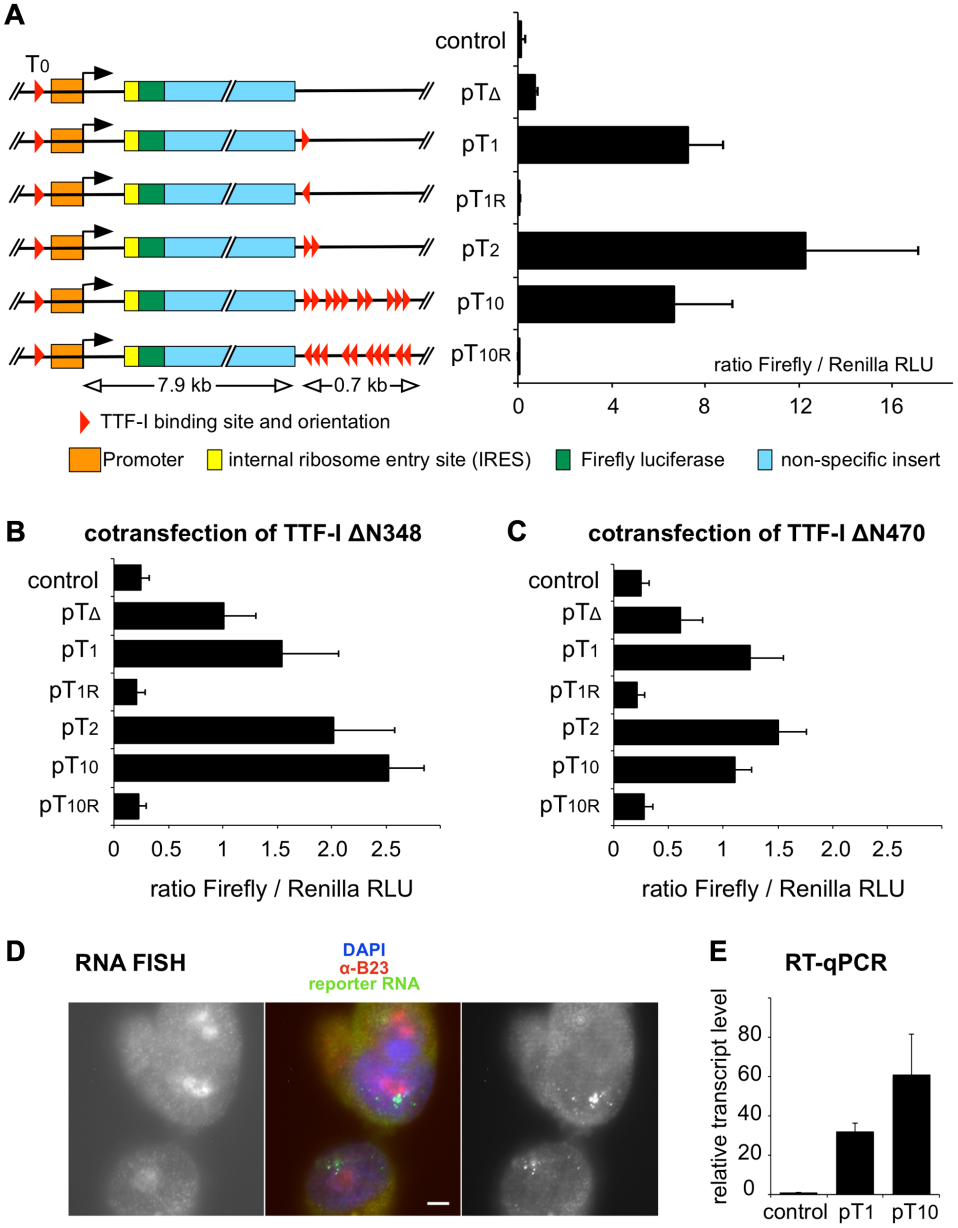
Multiple termination sites enhance transcription *in vivo*. (A) Reporter plasmids containing the rDNA promoter, *Firefly* luciferase and either no (pTΔ), one (pT_1_), two (pT_2_), ten (pT_10_) termination sites and T_1_ and T_1–10_ in reverse orientation (pT_1r_ and pT_10r_) were co-transfected with a *Renilla* luciferase encoding plasmid (pRL-TK) into CHO cells. As a control, empty pBluescript vector was co-transfected. Transcriptional activities were analysed using a dual luciferase reporter assay. The ratio *of Firefly*/*Renilla* relative light units (RLU) of three independent experiments is given. Error bars indicate standard deviations. The functional elements and the sizes of the reporter plasmids are depicted. (B) Reporter plasmids were co-transfected with a GFP-TTFΔN348 expression vector and analysed as described in (A). (C) Reporter plasmids were co-transfected with a GFP-TTFΔN470 expression vector and analysed as described in (A). (D) RNA FISH using CHO cell lines with stably integrated rDNA minigenes. CHO-pT_10_ cells containing an rDNA minigene with a full terminator, were stained with DAPI (in blue in the middle panel), with α-B23 antibody staining the nucleoli (left panel; shown in red in the middle panel), and integrated reporter gene transcripts were visualized by FISH (right panel; shown in green in the middle panel). Bar: 5 µm. (E) Transcription levels of genomically inserted pT_1_ and pT_10_ constructs were assayed using RT-qPCR. Comparative quantitation was performed and RNA levels of the *Firefly* luciferase sequence were normalized to β-actin expression. Relative transcript levels of three independent experiments are given in relation to non-transfected CHO Flp-In cells (control), error bars denote standard deviations.

To study the functional relevance of clustered sites *in vivo*, we transfected CHO cells with reporter plasmids containing the murine Pol I promoter, an internal ribosomal entry site (IRES), *Firefly* luciferase cDNA and either no terminator (pTΔ) or one (pT1), two (pT2) or ten (pT10) termination sites. As shown in Figure 3A, the presence of one or two terminators (pT1 and pT2) enhanced transcription of the luciferase reporter 8- to 12-fold compared to the terminator-deficient vector. The presence of ten termination sites (pT10) decreased luciferase activity, presumably due to squelching of endogenous TTF-I. In support of this view, transient overexpression of TTF-I (pTTFΔN348) revealed a linear correlation between the number of terminators and reporter gene activity, showing further transcriptional enhancement by the pT10 construct (Fig. 3B and Fig. S4B). Additional controls revealed that the stimulatory effect depends on the TTF-I binding sites at the promoter and the terminator (Fig. S4A) and co-transfection of pTTFΔN470 revealed that the chromatin-binding domain of TTF-I is required. pTTFΔN470 represents a deletion mutant that is capable of binding to its target sites on free DNA but not on chromatin [31]. Therefore, pTTFΔN470 cannot activate transcription in a chromatin context [31] and transfection of this construct did not further activate transcription of the pT10 construct (Fig. 3C and Fig. S4B). The control shows that chromatin-specific activities of TTF-I are required for efficient transcriptional activation. Notably, there was no luciferase expression using reporters with TTF-I binding site(s) in the reverse orientation (pT1R, pT10R), supporting the importance of the topological arrangement of the HCTs for efficient Pol I transcription.

To examine whether the number of terminators affects gene activity and/or the spatial organization of rDNA in a genomic context, we generated stable cell lines that harbour a single copy of mouse rRNA minigenes, either containing only T_1_ (CHO-pT1) or all ten terminators (CHO-pT10) (Fig. S5). Using the Flip-In system we generated comparable rRNA minigene lines, integrated at the same genomic site of CHO cells. This strategy allows us to rule out effects of inefficient chromatin packaging and minigene dosage in transfection experiments. The nuclear localisation of the ectopic rDNA was not affected, as 3D immuno-FISH experiments revealed that comparable number of rDNA was associated with the nucleoli in the stable cell lines (33 of 104 alleles were associated with nucleoli in CHO-pT1 and 50 of 160 alleles in CHO-pT10 cells; Fig. S5A, B). RNA FISH experiments confirmed that all cell lines were transcriptionally active (Fig. 3D). Expression analysis of the rRNA minigene by qRT-PCR and reporter assays revealed that both the level of the ectopic pre-rRNA and the Pol I-driven luciferase activity were increased in CHO-pT10 compared to CHO-pT1 cells (Fig. 3E and Fig. S5C), reinforcing the activating role of clustered termination sites in rDNA transcription.

### HTC is required for gene looping and efficient loading of Pol I specific factors

**Figure 4:**
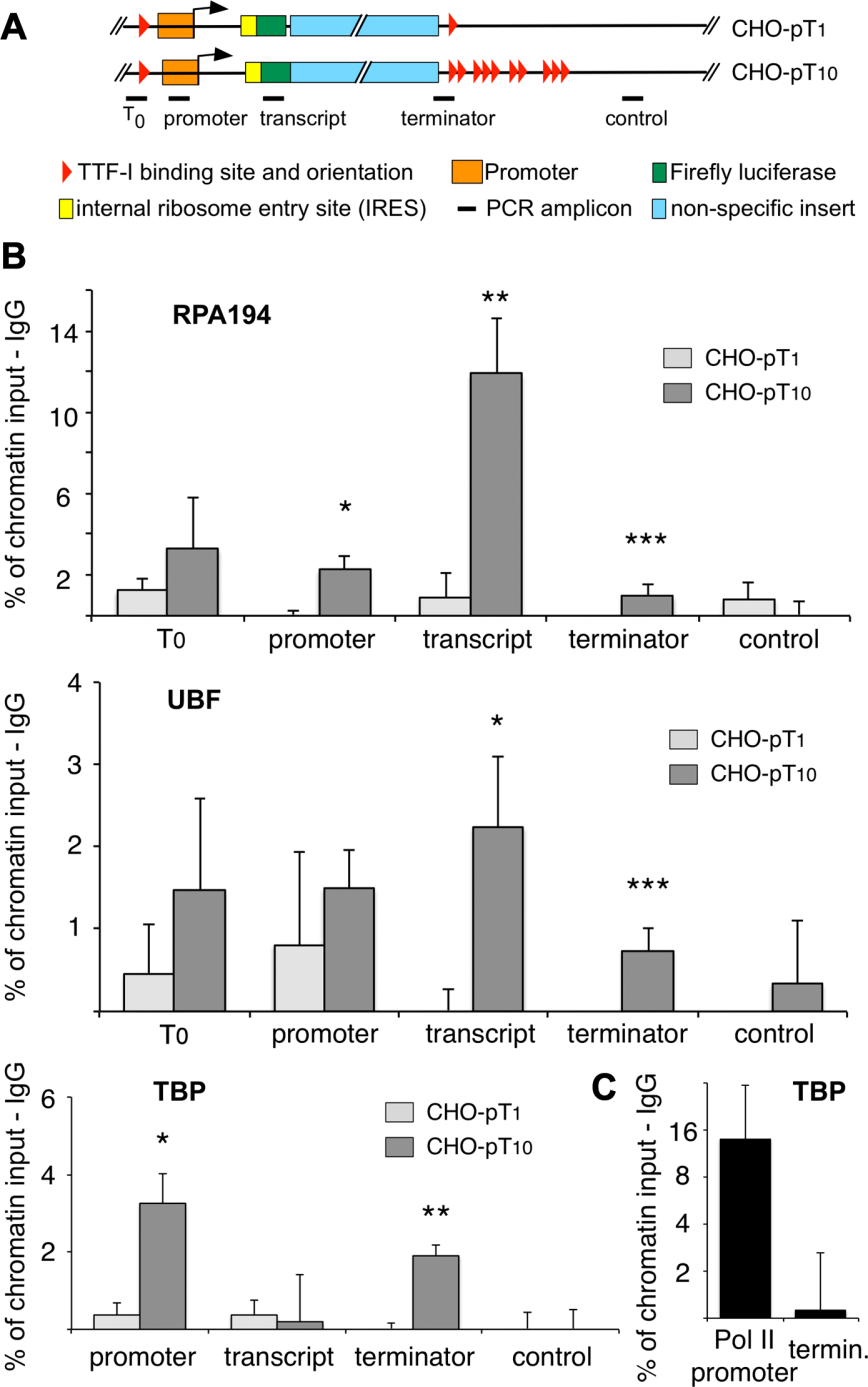
Clustered termination sites enhance transcription and are required for chromatin looping. (A) Overview to the stably integrated rDNA minigenes and the locations of the PCR amplicons. (B) Chromatin-immunoprecipitation (ChIP) assays on stably integrated rDNA reporter genes using the indicated antibodies. Occupancies were measured by qPCR, calculated as percentage of input chromatin and background signals as determined from control IPs with unspecific antibodies (α-HA or α-IgG) were subtracted. At least three independent biological replicates were performed. Error bars indicate the standard error of the mean. For statistical analysis, a two-sided, homoscedatic student's t-test was performed, stars denote significances. * p<0.05, ** p<0.01, *** p< = 0.001. (C) ChIP experiment using an rDNA reporter in which the Pol I spacer promoter, core promoter and enhancer regions of a pT_10_ reporter construct were replaced by a Pol II promoter containing a canonical TBP binding site. The experiment was performed as described in (B).

To decipher the molecular mechanism underlying HTC-driven transcriptional activation, we compared transcription factor occupancy within the stable cell lines, containing single rDNA minigenes with either one (CHO-pT1) or ten terminators (CHO-pT10, Fig. 4A). As shown in Figure 4B, binding of Pol I and UBF was enhanced at the promoter, the transcribed region and the terminators of CHO-pT10 compared to CHO-pT1 cells. In addition, we observed increased binding of TBP to the promoter of CHO-pT10 cells, demonstrating that augmented rDNA transcription is a direct consequence of enhanced transcription initiation and polymerase occupancy. Pol I enrichment downstream of the terminator region was reduced in CHO-pT10 cells, consistent with clustered TTF-I binding sites promoting efficient termination. Similar results were obtained with the transient transfection of the constructs (Fig. S6).

Active rRNA genes are known to form chromatin loops, connecting the promoter with the terminator to promote recycling of Pol I [22], [23], [33]. To examine whether multiple terminators facilitate loop formation, we determined the occupancy of TBP at the terminator in the stable cell lines CHO-pT1 and CHO-pT10 (Fig. 4B lower panel and 4C). The close proximity of a protein to DNA results in crosslinking and co-purification of the DNA, even though the factor does not directly contact the DNA at this site. Such binding events indicate the close spatial proximity of distant DNA sites, comparable to 3C assays [34]. Obviously, TBP was found to be associated with the promoter of CHO-pT1 and CHO-pT10 as part of the initiation complex, while no binding was observed in the transcribed region (Fig. 4B, TBP panel). Strikingly, TBP was also enriched at the terminator of CHO-pT10 but not CHO-pT1 cells, suggesting that clustered TTF-I binding sites are in close proximity with the gene promoter. Consistent with multiple terminators facilitating initiation of transcription, TBP and Pol I occupancy was about 4-fold higher in CHO-pT10 compared to CHO-pT1 (Fig. 4B, TBP panel). To exclude the possibility that clustered TTF-I binding sites on their own recruit TBP to the 3′-end of rRNA genes, we examined TBP occupancy on a reporter plasmid in which the ten terminators were fused to a Pol II promoter. TBP was enriched at the Pol II promoter but close to background at the terminator (Fig. 4C), emphasizing the importance of TTF-I binding sites at both elements, the promoter and the terminators, to form chromatin loops.

### Integrative analysis of histone marks reveals similarity to classical enhancer elements

**Figure 5:**
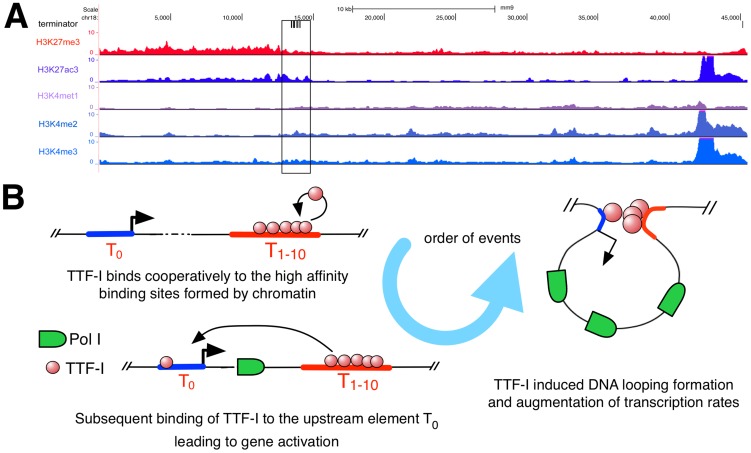
Distribution of histone modifications at the murine rDNA. (A) Enrichment of histone modifications at the rDNA locus in 3T3-L1 cells. The whole rDNA repeat is plotted from position +1 (the TSS) to position 45.309. The terminator track indicates TTF-I binding sites by black vertical lines. The black box highlights the clustered terminator elements at the 3′ end of the gene. ChIP-Seq tracks of histone modifications display relative enrichments compared to input. (B) Model depicting the order of binding events at the rRNA gene. The promoter is coloured in blue, a right-headed arrow marks the TSS and the clustered termination sites are depicted in red.

Homo- and heterotypic clusters of transcription factor binding sites were shown to mark potential regulatory regions with enhancer function [35]–[39] characterized by eukaryotic histone marks like H3K27ac, which is involved in long-range chromatin interactions [40]. As the repetitive rDNA is left out of standard ChIP-Seq analyses, we artificially added a single mouse rDNA repeat to the current mouse genome version mm9 and mapped ChIP-Seq data of H3K27ac, H3K27me3, H3K4me1, H3K4me2 and H3K4me3 to this expanded reference genome (Fig. 5A). We observed enrichment of H3K27ac and H3K4me2 in the terminator and promoter region of murine rDNA, enforcing our previous results and confirming that the homotypic cluster of TTF-I binding sites represents an active enhancer element. In contrast, H3K27me3, commonly associated to repressed genes, is depleted at the terminator compared to the rDNA gene body. Therefore, the mouse rDNA terminator exhibits a histone modification profile typical for enhancer elements involved in Pol II transcription.

## Discussion

Clustering of transcription factor binding sites, comprising either multiple binding sites for the same factor (homotypic clustering) or different DNA binding motifs (heterotypic clustering), is an important regulatory feature of eukaryotic gene expression, about 62% of transcription factor genes and 66% of developmentally regulated genes comprising clustered binding sites in vertebrates [3]. Therefore, this feature has been widely used for computational prediction. In *Drosophila*, predicted HTCs are present in more than 70% of regulatory regions and have been suggested to function as developmental enhancers [6], [41]. Clustered binding sites are suggested to exert a positive effect on transcription by either of the following mechanisms. They could increase the local concentration of transcription factors or facilitate multiple interactions with components of the transcription machinery. Alternatively, they could provide functional redundancy [37], [42], allowing cooperative binding of the factors through interactions among the multiple binding sites or indirectly through multiple interactions with the transcriptional machinery [10], [43]–[46]. Here, we have uncovered a novel chromatin-based mechanism underlying HTC-directed transcriptional activation. We show that packaging into chromatin converts multiple low-affinity terminators downstream of the rDNA transcription unit into a high-affinity binding platform for TTF-I. This preferential binding of TTF-I to the downstream terminators is a prerequisite for TTF-I binding to the promoter-proximal binding site, connecting the promoter with the terminator to allow efficient recycling of Pol I.

Cooperative binding of proteins has been shown to disrupt nucleosomes, thereby increasing the accessibility of transcription factors to regulatory sites [47], [48]. Our data reveal an alternative mechanism that increases the affinity of transcription factors. We show that binding of TTF-I to its target sites in chromatin is higher than to free DNA, suggesting that a specific nucleosomal arrangement or interactions with histones may trigger cooperative binding of TTF-I. Thus, HTCs attract transcription factors to functionally relevant sites, avoiding binding to single target sites in the genome. High-affinity binding of TTF-I to clustered termination sites will ensure loading of the downstream terminators (T_1_–T_10_) prior to TTF-I binding to the promoter-proximal binding site T_0_ *in vivo*. Sequential binding of TTF-I to the 3′- and 5′-end of the rDNA transcription unit will ensure that transcription initiation will take place exclusively at rRNA genes that are associated with TTF-I and will be properly terminated. In addition, a direct interaction between the promoter and the terminator is only established when the terminator comprises several TTF-I binding sites. This mode of binding and the formation of an intragenic loop may serve two functions. First, it links the terminator with the respective transcription unit to be activated. Second, it enhances transcription at genes associated with TTF-I by forming a highly active ribomotor structure [22], [49]. Thus, homotypic clustering of TTF-I binding sites coordinates transcription initiation and termination, thereby affecting both the timing and the efficiency of rDNA transcription.

It is well established that gene activation by a distal regulatory element correlates with long-range interactions between enhancer(s) and gene promoters by factor-mediated formation of chromatin loops [50]. With regard to human and rat rRNA genes, previous studies suggested a role for TBP and c-Myc in loop formation at active rRNA genes [23], [33]. However, genome-wide ChIP-Seq data did not reveal significant enrichment of c-Myc- and TBP at the terminator (Fig. S7B). Moreover, murine rRNA genes lack clustered E-boxes (Fig. S7A), and therefore the participation of c-Myc in loop formation is not very likely. Similar loop mechanisms were shown for RNA polymerase II transcribed genes, suggesting a common theme involving the interaction of promoters with transcription termination regions that enhance the transcriptional activity and gene regulation [51].

Active enhancer elements are characterized by eukaryotic histone marks, e.g. H3K27ac or H3K4me1, which are involved in long-range chromatin interactions [40]. Notably, our integrative genomic analysis revealed characteristic enrichment of histone marks at the terminator, which can be observed in human as well [52]. The results support our finding that the homotypic cluster of TTF-I binding sites displays all hallmarks of a functional enhancer, such as distal location, presence of HTCs, regulatory histone marks and the potential to exert gene activation by direct, protein-mediated DNA loops. Chromatin-dependent high-affinity binding of TTF-I to the clustered binding sites adds a further regulatory level on the enhancer function, i.e., coordination of transcription termination and initiation.

## Materials and Methods

### Protein expression and microscale thermophoresis

Histidine-tagged full-length TTF-I and the deletion mutants TTFΔN210 and TTFΔN348 were purified on a heparin column (Bio-Rad), followed by purification with Ni-NTA agarose according to the manufacturer's instructions (Qiagen). For microscale thermophoresis experiments, 50 nM of fluorescently labelled DNA oligonucleotides were incubated with 5 nM–2.4 µM of protein for 10 min at 30°C in 80 mM Tris-HCl (pH 7.6), 80 mM KCl, 0.2 mM EDTA, 5 mM MgCl_2_, 10% glycerol and 0.05% IGEPAL CA-630. Affinity measurements were carried out in a Monolith NT.015T (NanoTemper Technologies) as described [26].

### MNase footprinting and transcription

300 ng of chromatin reconstituted with *Drosophila* extract was digested with 1.5 U of MNase (Sigma) for 40 s in 10 mM Tris-HCl (pH 7.6), 80 mM KCl, 1.5 mM MgCl_2_, 10% glycerol, 0.5 mM ATP, and 200 ng/µl BSA. Reactions were then stopped by the addition of 0.2 volumes of 4% SDS, 100 mM EDTA, 1 µg of glycogen, 10 µg of proteinase K. Purified DNA was analysed by a single round of PCR (denaturation, 5 min at 95°C; annealing, 2 min at 56°C; extension, 1 min at 72°C) using radioactively labelled oligonucleotides that hybridize to the rDNA promoter or terminator. Primer extension fragments were resolved on 8% sequencing gels and visualized by autoradiography.

Transcription experiments were performed on pMrWT-T, a template comprising the murine rDNA promoter (from −170 to +155 with regard to the transcription start site) fused to a 3.5 kb 3′-terminal rDNA fragment (BamHI/EcoRV Fragment) harbouring all ten terminators. (T_1_–T_10_). The promoter and the terminator elements are separated by 686 bp. Transcription reactions were performed as described [53].

### Cell culture

CHO and CHO Flp-In cells (Invitrogen) were grown in DMEM (GIBCO) supplemented with 10% FBS, 100 U/ml penicillin and 100 µg/ml streptomycin. For transient transfections, 200.000 cells were transfected with 1 µg of plasmid DNA. Prior to transfection of the CHO Flp-In cells, 100 µg/ml zeocin (Invitrogen) was added to the medium and for transfection 0.25, 0.5 or 1.0 µg of the rRNA reporter construct and the flipase encoding plasmid pOG44 (Invitrogen) in a ratio of 1∶9 were used. During the selection process, 500 µg/ml of hygromycin (PAA) was added to the medium; afterwards the stable cell lines were passaged with 250 µg/ml of hygromycin.

### Constructs and reporter gene assays

Transiently transfected rRNA minigenes [22] contain mouse rDNA (BK000964) sequences from position −1932 to +181, an IRES, the firefly luciferase gene, and rDNA terminator regions from position +13169 to +15278 (T_10_ constructs) in a pGL3-Basic vector (Promega). Plasmids for genomic integration contain in addition the enhancer/promoter regions from position −2148 to +181 cloned into a pcDNA5-FRT vector (Invitrogen).

Cells were transfected with Pol I driven firefly luciferase reporter constructs and a Pol II renilla luciferase control plasmid, pRL-TK (Promega). TTF-I co-transfections were performed with the expression vectors TTFΔN348-EGFP or TTF-IΔN470-EGFP in a TTF-I∶reporter ratio of 10∶1. Protein expression was monitored by Western Blot analysis. Reporter gene measurements were performed using the Dual Luciferase Reporter Assay System (Promega) according to the manufacturer's instructions using a single-tube luminometer (Stratec Biomedical Systems).

### Isolation of RNA and genomic DNA

RNA isolation was performed with the NucleoSpin RNA II kit (Macherey-Nagel). Purified RNA (500 ng) was used for cDNA preparation with the iScript Select kit (Biorad).

To determine the number of integration sites, genomic DNA was isolated by cells lysis (1% SDS, 50 mM Tris-HCl (pH 8.0), 20 mM EDTA and 250 µg of RNase A), the addition of proteinase K and incubation at 37°C o.n. The supernatant was precipitated with ethanol and ammonium acetate.

### qPCR

Quantitative real-time PCR was performed in a Rotor-Gene cycler (Qiagen) using a HotStar master mix containing SYBR green (Qiagen). Primer sequences and annealing temperatures are listed in the in Table S2. Fold inductions were calculated using the comparative quantitation software (Qiagen). Post-PCR melting curves and agarose gels of PCR products (Fig. S3F) were used to assess the quality of primer pairs.

### Chromatin immunoprecipitation

Cells were transfected with 10 µg of DNA and cross-linked with 1% formaldehyde for 10 min (α-Pol I and α-UBF ChIPs) or 10 mM DMA for 30 min +1% formaldehyde for 10 min (α-TBP) at RT. The reactions were quenched with 125 mM glycine. Cells were washed twice in ice-cold PBS and the cell pellets were lysed in SDS lysis buffer (1% SDS, 50 mM Tris-HCl pH 8.0, 20 mM EDTA, protease inhibitors). Chromatin was sheared in a Biorupter sonicator (Diagenode) to fragments of 400–1000 bp in length. The samples were diluted in IP dilution buffer (20 mM Tris-HCl, 2 mM EDTA, 1% Triton X-100, 150 mM NaCl, pH 8.0, protease inhibitors). Paf53 antibody for Pol I detection and the pre-serum were obtained from the Grummt lab [54]. Antibodies targeting RPA194 (sc-28714), UBF (sc-9131), TBP (sc-273) and normal rabbit IgG (sc-2027) were purchased from Santa Cruz. Antibodies (5 µg) and chromatin were incubated on a rotating wheel at 4°C o.n. Pre-blocked Protein-G sepharose (500 µg/ml sonicated salmon sperm DNA and 100 µg/ml BSA in IP dilution buffer) was added to isolate the immune-complexes and incubated for 2 h at 4°C. Beads were washed twice with IP dilution buffer, once with high salt buffer (20 mM Tris-HCl, 2 mM EDTA, 1% Triton X-100, 150 mM NaCl, pH 8.0), LiCl buffer (0.25 M LiCl, 1% NP40, 1% Deoxycholate, 1 mM EDTA, 10 mM Tris-HCl, pH 8.0) and twice with TE buffer (10 mM Tris-HCl, 1 mM EDTA pH 8.0). Elution was performed using 250 µl of 1% SDS, 0.1 M NaHCO_3_. RNase A was added to a concentration of 100 µg/ml and incubated for 2.5 h at 37°C. Following the Proteinase K digestion (100 µg/ml, 2.5 h at 37°C), reverse crosslinking was carried out at 64°C o.n. DNA was isolated by phenol/chloroform/isoamylalcohol extraction and precipitated with ethanol and sodium acetate.

### FISH experiments

Fluorescence in situ hybridizations on metaphase chromosome spreads and on interphase nuclei combined with nucleolar immunostaining were performed as described [55]. For RNA FISH, cells grown on coverslips were fixed for 10 min at room temperature with 3.7% formaldehyde/5% acetic acid/0.5% (w/v) NaCl, washed twice with 1× PBS, once in 50 mM NH_4_Cl/1× PBS pH 7.4, and once in 1× PBS. Coverslips were then transferred to 70% ethanol and incubated o.n. at 4°C. Before hybridization, coverslips were rehydrated in 2× SSC/50% formamide for 15 min at RT. Hybridization mixtures were added for o.n. incubation at 37°C. Post-hybridization washes were carried out as follows: 2×25 min at 37°C in 50% formamide/2× SSC and 2×5 min in 2× SSC at RT. The subsequent immunostaining, DNA staining and mounting was performed as in interphase DNA FISH experiments. Nick-translated, biotin-labeled pcDNA5-FRT-rRNA reporter served as hybridization probe in all experiments.

### Visualisation of histone modification data at the mouse rDNA locus

A custom build of the mm9 assembly was generated by replacing unsequenced bases at the 5′-end of chromosome 18 with a murine rDNA repeat (GenBank accession no. BK000964). We used Bowtie [56] to align published ChIP-seq data sets of 3T3L1 and MEL cells (for details see Table S1) to the custom assembly using ‘–best -k 1’ settings. Input-normalized bedGraph files were generated using the makeUCSCfile.pl script contained in the HOMER software suite (http://biowhat.ucsd.edu/homer/, [57]) using standard settings.

## Supporting Information

Figure S1 Related to Figure 1. Clustering of rRNA gene termination sites is evolutionary conserved. (A) Distribution of binding sites being involved in transcription termination of mouse and human rRNA genes. The relative distance to the end of the coding region and the distances between the individual binding sites are given. Lollipops mark TTF-I binding sites. Sequence comparison of the TTF-I binding sites in mouse and human is shown below. (B) MNase digestion of reconstituted chromatin. Chromatin was reconstituted with the *Drosophila* embryo extract and digested with increasing amounts of MNase. Purified DNA was visualized by agarose gel electrophoresis and ethidium bromide staining. The regular fragment ladder is indicative of an efficiently assembled nucleosomal array (1n through 8n).
(JPG)

Figure S2 Related to Figure 1. Multiple termination sites are required for efficient transcription activation. (A) *In vitro* transcription analysis was performed comparatively on free DNA (lanes 1–5) or *in vitro* assembled chromatin (lanes 6–10) on pMrSB containing a single termination site (T_1_), either in the absence (lanes 1 and 6) or presence of TTF-I (lanes 2–5 and 7–10). The radioactively labeled transcripts were separated by PAA gel electrophoresis and detected by autoradiography. (B) *In vitro* transcription using the rRNA minigene pMrBH harboring the first two termination sites (T_1_+T_2_). The DNA was analysed for *in vitro* transcription on free DNA and chromatin with increasing amounts of TTF-I as described in (A). (C) *In vitro* transcription using the rRNA minigene pMrT5 harbouring the first five termination sites (T_1_ to T_5_). The DNA was analysed for *in vitro* transcription on free DNA and chromatin with increasing amounts of TTF-I as described in (A).
(JPG)

Figure S3 Related to Figure 2. TTF-I binds with higher affinity to the rDNA terminator in reconstituted chromatin. (A) Overview to the experimental strategy. The plasmid pMrEnLT10 containing the gene promoter, a 5 kb long transcribed region and the full terminator region was used for TTF-I binding experiments. Specific primers for PCR amplification of the regions containing T_0_ (Promoter, P, 145 bp), T_1_ to T_3_ (Terminator, T, 276 bp) and a control region of the vector (control, c, 187 bp) were designed. Primers were mixed to allow simultaneous detection and quantification of the three DNA regions. The plasmid was used as free DNA or reconstituted into chromatin with the *Drosophila* embryo extract. DNA or chromatin was incubated with TTF-I for 10 min and then partially digested with MNase (50 fmoles of DNA were incubated with 2 U MNase for 20 s; 300 ng of chromatin was incubated with 50 U MNase for 30 s; the reactions were stopped by the addition of EDTA to a final concentration of 5 mM). TTF-I bound DNA fragments were retained on Ni-NTA material in a batch assay and washed twice in Ex150 buffer. The associated DNA was purified and analysed by PCR using the mixture of primers. (B) Binding of TTF-I to the promoter and the terminator on free DNA. 50 fmoles of free DNA were incubated with increasing amounts of TTF-I (60 fmol to 4 pmol, lanes 6 to 12) and DNA was partially hydrolysed with MNase. A control digestion revealing the input DNA is shown in lane 14. Purified DNA was amplified with a mixture of primers giving rise to the Promoter (P), Terminator (T) and control (c) PCR fragments. Lanes 1 to 4 show the PCR amplification of increasing amounts of the partially digested pMrEnLT10 plasmid, revealing that the individual fragments were amplified with similar efficiency over a 16-fold concentration difference. Ni-NTA purification of the DNA in the absence of TTF-I gives rise to a background of PCR fragments (lane 5) that remains in the fractions containing increasing amounts of TTF-I (lanes 6 to 12). However, with higher concentrations of TTF-I the promoter and terminator fragments accumulated with similar efficiency (250 fmoles to 4 pmoles, lanes 8 to 12) suggesting binding of TTF-I. The promoter and terminator fragments appear with similar TTF-I concentrations, suggesting similar binding affinities of TTF-I with the promoter and terminator sites on free DNA. (C) Binding of TTF-I to the promoter and the terminator in reconstituted chromatin. A control digestion of chromatin revealing the input DNA is shown in lane 15. The same experiment as shown in B) was performed with reconstituted chromatin. Lanes 1 to 4 show the PCR amplification of increasing amounts of the partially digested pMrEnLT10 plasmid reconstituted into chromatin. Incubation of chromatin with increasing concentrations of TTF-I (62, 125, 250, 500, 2000, 4000 fmol, lanes 6 to 11) revealed an amplification of the terminator fragment at lower TTF-I concentrations (starting in lane 7) than for the promoter fragment (starting in lane 10). The result suggests that TTF-I binds with higher affinity to the rDNA terminator reconstituted into chromatin and with lower affinity to the chromatinized rDNA promoter. The result confirms the *in vitro* transcription experiment (Figure 1) and the MNase footprinting data (Figure 2).
(JPG)

Figure S4 Related to Figure 3. Promoter-proximal and terminator TTF-I binding sites and the transactivation domain of TTF-I are required for full transcriptional activation of rRNA minigenes *in vivo*. (A) Transiently transfected rRNA minigenes contain mouse rDNA (BK000964) sequences from position −217 (pT_10_s, pTΔs) or −148 (pΔT_0_T_10_s) to +181, an IRES, the *Firefly* luciferase gene, and rDNA terminator regions from position +13169 to +15278 (pΔT_0_T_10_s and pT_10_s) in a pGL3-Basic vector (Promega). The plasmids contain a shorter non-specific insert than the constructs shown in Figure 3A. The insert size is 3 kilobases between the promoter and terminator region. CHO (left panel) or NIH3T3 cells (right panel) were transfected with Pol I driven *Firefly* luciferase reporter constructs and a Pol II *Renilla* luciferase control plasmid, pRL-TK (Promega). Reporter gene measurements were performed using the Dual Luciferase Reporter Assay System (Promega). Deletion of either the promoter-proximal or the terminator TTF-I binding sites reduces transcriptional activity, complementing the results shown in Figure 3. (B) Western Blot of transiently transfected CHO cells expressing EGFP-tagged TTF-I deletion mutants used in Figure 3B and C. Detection was performed with an α-GFP (sc-8334) and subsequently an α-TTF-I antibody (αC7). Lane 2: control transfection with a vector expressing only EGFP, lanes 3–4: overexpressed EGFP-tagged TTF-I ΔN348 or TTF-I ΔN470, lane 5: non-transfected control CHO cells. Endogenous full-length TTF-I is visible in all lanes. MW = molecular weight marker.
(JPG)

Figure S5 Related to Figure 4. Characterization of stable cell lines containing a single mouse rRNA gene. rRNA minigenes containing one or ten termination sites (pT_1_ and pT_10_) were genomically inserted into CHO Flp-In cells and stable single integrants were selected. This resulted in the cell lines CHO-pT_1_ and CHO-pT_10_. In all experiments, non-transfected CHO Flp-In cell lines were used as controls. Bars represent the mean of three independent stable transfections and error bars indicate standard deviations. (A) FISH detection of genomically inserted mouse rRNA minigenes on CHO Flp-In metaphase spreads. Chromosomes were stained with DAPI and are illustrated in red. Hybridization signals of reporter probes are shown in green. Arrows indicate the single genomic insertion site. The lower panel shows copy number determination of the integrated rDNA reporter plasmids. qPCR was performed on genomic DNA and comparative quantitation was performed between the luciferase gene and the copy number of two single-copy housekeeping genes, β-actin and PabpnI. Bars represent the mean of two independent experiments, error bars denote standard deviations. (B) The number of termination sites does not influence localization of the rDNA minigenes. 3D immuno-FISH analysis of genomically inserted pT_1_ and pT_10_ in interphase nuclei. Nuclear DNA was stained with DAPI (shown in blue in the middle merged panel), nucleoli with an α-B23 antibody and indirect immunofluorescence (left panel, and shown in red in the middle merged panel), and the rRNA minigenes were visualized by FISH (right panel, and shown in green in the middle merged panel). Bars depict the percentage of genomically integrated minigenes associated to the nucleolus, n denotes the absolute number of assayed alleles. Bar: 5 µm. (C) *Firefly* luciferase reporter gene assay on genomically integrated rRNA minigenes. Relative light units (RLU) were measured in three independent experiments, error bars indicate standard deviations. As control, non-transfected CHO Flp-In cells were assayed.
(JPG)

Figure S6 Related to Figure 4. ChIP experiments in transiently transfected CHO cells. (A) Overview of rDNA minigenes and the locations of the PCR amplicons. (B) Chromatin-immunoprecipitation (ChIP) assays on transiently transfected rDNA reporter genes using the indicated antibodies. Occupancies were measured by qPCR, calculated as percentage of input chromatin and background signals as determined from control IPs with unspecific antibodies (α-IgG or α-HA Tag) were subtracted. Three independent biological replicates were performed. Error bars indicate the standard error of the mean. (C) Sonication test. Representative agarose gel of the chromatin input sonicated for 5 or 10 min (30 sec on/30 sec off, settings: “high”) after proteinase K digestion and reversal of crosslinking. 10 min sonication time was used for all experiments. Fragment size range: 100–600 bp. MW = molecular weight marker. (D) Representative agarose gel of qPCR amplicons, pipetted in duplicates, after 40 cycles of qPCR. MW = molecular weight marker. (E) Mouse-specific primer pairs were tested on non-transfected CHO cells to ensure species-specific amplicons. Chromatin was isolated from CHO cells, processed like an input for ChIP experiments and analysed by qPCR. DNA levels were normalised to the 5′ IGS signal of hamster rDNA (5′ IGS). The multi-copy rRNA genes show a 25-fold higher signal than the single-copy gene β-actin. None of the mouse specific primer pairs amplifies detectable products on hamster chromatin. A faint signal appears in the plasmid-specific control primer pair. Each bar represents the mean of three replicates. For every primer pair, both CHO chromatin template triplicates (left) and water control (right) are shown.
(JPG)

Figure S7 Related to Figure 5. Distribution E-boxes, c-Myc and TBP at the murine rDNA. (A) *In silico* comparison of the human and mouse rDNA repeat. The murine terminator region comprising of T_1_ to T_10_ does not overlap with E-box elements, the canonical c-Myc binding sites. (B) Enrichment of histone modifications at rDNA in MEL cells. The whole rDNA repeat is plotted from position +1 (the TSS) to position 45.500. The terminator track indicates TTF-I binding sites by black vertical lines. The black box highlights the clustered terminator elements at the 3′ end of the gene. ChIP-Seq tracks of c-Myc and TBP display relative enrichments compared to input.
(JPG)

Table S1 Summary of published NGS data used in this study. The table provides an overview of all next-generation sequencing datasets that have been used in the study. Cell types, accession numbers and respective publications are indicated for each dataset. The number of reads indicates absolute tag counts of sequencing reads mapped to the expanded reference genome.
(DOC)

Table S2 List of qPCR primers used for ChIP analyses. The primer lists contains all primers used for quantitation of ChIP assays. Name, binding site, sequence and annealing temperatures are provided for each primer pair used in the study.
(DOCX)

## References

[1] Berg OG, Hippel von PH (1987) Selection of DNA binding sites by regulatory proteins. Statistical-mechanical theory and application to operators and promoters. Journal of Molecular Biology 193: 723–750. 3612791 10.1016/0022-2836(87)90354-8

[2] Berg OG, Hippel von PH, Hippel von PH (1988) Selection of DNA binding sites by regulatory proteins. Trends in Biochemical Sciences 13: 207–211. 3079537 10.1016/0968-0004(88)90085-0

[3] Gotea V, Visel A, Westlund JM, Nobrega MA, Pennacchio LA, et al (2010) Homotypic clusters of transcription factor binding sites are a key component of human promoters and enhancers. Genome Research 20: 565–577. 20363979 10.1101/gr.104471.109 PMC2860159

[4] Davidson EH (2002) A Genomic Regulatory Network for Development. Science 295: 1669–1678. 11872831 10.1126/science.1069883

[5] Schroeder MD, Pearce M, Fak J, Fan H, Unnerstall U, et al (2004) Transcriptional Control in the Segmentation Gene Network of Drosophila. PLoS Biol 2 (9) e271. 15340490 10.1371/journal.pbio.0020271 PMC514885

[6] Erives A, Levine M (2004) Coordinate enhancers share common organizational features in the Drosophila genome. Proc Natl Acad Sci USA 101: 3851–3856. 15026577 10.1073/pnas.0400611101 PMC374333

[7] Rhee HS, Pugh BF (2011) Comprehensive Genome-wide Protein-DNA Interactions Detected at Single-Nucleotide Resolution. Cell 147: 1408–1419. 22153082 10.1016/j.cell.2011.11.013 PMC3243364

[8] Sauer F, Hansen SK, Tjian R (1995) Multiple TAFIIs directing synergistic activation of transcription. Science 270: 1783–1788. 8525367 10.1126/science.270.5243.1783

[9] Sauer F, Hansen SK, Tjian R (1995) DNA template and activator-coactivator requirements for transcriptional synergism by Drosophila bicoid. Science 270: 1825–1828. 8525377 10.1126/science.270.5243.1825

[10] Hertel KJ, Lynch KW, Maniatis T (1997) Common themes in the function of transcription and splicing enhancers. Current Opinion in Cell Biology 9: 350–357. 9159075 10.1016/s0955-0674(97)80007-5

[11] Vashee S, Melcher K, Melcher K, Ding WV, et al (1998) Evidence for two modes of cooperative DNA binding in vivo that do not involve direct protein-protein interactions. Current biology : CB 8: 452–458. 9550700 10.1016/s0960-9822(98)70179-4

[12] Grummt I, Rosenbauer H, Niedermeyer I, Maier U, Ohrlein A (1986) A repeated 18 bp sequence motif in the mouse rDNA spacer mediates binding of a nuclear factor and transcription termination. Cell 45: 837–846. 3458534 10.1016/0092-8674(86)90558-1

[13] Grummt I, Maier U, Ohrlein A, Hassouna N, Bachellerie JP (1985) Transcription of mouse rDNA terminates downstream of the 3″ end of 28S RNA and involves interaction of factors with repeated sequences in the 3″ spacer. Cell 43: 801–810. 4075406 10.1016/0092-8674(85)90253-3

[14] La Volpe A, Simeone A, Simeone A, D'Esposito M, et al (1985) Molecular analysis of the heterogeneity region of the human ribosomal spacer. Journal of Molecular Biology 183: 213–223. 2989541 10.1016/0022-2836(85)90214-1

[15] Bartsch I, Schoneberg C, Grummt I (1987) Evolutionary changes of sequences and factors that direct transcription termination of human and mouse ribsomal genes. Molecular and Cellular Biology 7: 2521–2529. 3649563 10.1128/mcb.7.7.2521-2529.1987 PMC365386

[16] Clos J, Normann A, Ohrlein A, Grummt I (1986) The core promoter of mouse rDNA consists of two functionally distinct domains. Nucleic Acids Research 14: 7581–7595. 3774539 10.1093/nar/14.19.7581 PMC311782

[17] Strohner R, Nemeth A, Nemeth A, Jansa P, et al (2001) NoRC–a novel member of mammalian ISWI-containing chromatin remodeling machines. The EMBO Journal 20: 4892–4900. 11532953 10.1093/emboj/20.17.4892 PMC125270

[18] Santoro R, Li J, Grummt I (2002) The nucleolar remodeling complex NoRC mediates heterochromatin formation and silencing of ribosomal gene transcription. Nature Genetics 32: 393–396. 12368916 10.1038/ng1010

[19] Yuan X, Feng W, Imhof A, Grummt I, Zhou Y (2007) Activation of RNA Polymerase I Transcription by Cockayne Syndrome Group B Protein and Histone Methyltransferase G9a. Molecular Cell 27: 585–595. 17707230 10.1016/j.molcel.2007.06.021

[20] Xie W, Ling T, Zhou Y, Feng W, Zhu Q, et al (2012) The chromatin remodeling complex NuRD establishes the poised state of rRNA genes characterized by bivalent histone modifications and altered nucleosome positions. Proceedings of the National Academy of Sciences 109: 8161–8166. 10.1073/pnas.1201262109 PMC3361413 22570494

[21] Sander EE, Grummt I (1997) Oligomerization of the transcription termination factor TTF-I: implications for the structural organization of ribosomal transcription units. Nucleic Acids Research 25: 1142–1147. 9092622 10.1093/nar/25.6.1142 PMC146573

[22] Németh A, Guibert S, Tiwari VK, Ohlsson R, Längst G (2008) Epigenetic regulation of TTF-I-mediated promoter–terminator interactions of rRNA genes. The EMBO Journal 27: 1255–1265. 18354495 10.1038/emboj.2008.57 PMC2367401

[23] Denissov S, Lessard F, Mayer C, Stefanovsky V, van Driel M, et al (2011) A model for the topology of active ribosomal RNA genes. EMBO reports 12 (3) 231–7 doi:10.1038/embor.2011.8 21331097 10.1038/embor.2011.8 PMC3059908

[24] Németh A, Längst G (2011) Genome organization in and around the nucleolus. Trends in Genetics 27: 149–156. 21295884 10.1016/j.tig.2011.01.002

[25] Gerber JK, Gögel E, Berger C, Wallisch M, Müller F, et al (1997) Termination of mammalian rDNA replication: polar arrest of replication fork movement by transcription termination factor TTF-I. Cell 90: 559–567. 9267035 10.1016/s0092-8674(00)80515-2

[26] Zillner K, Jerabek-Willemsen M, Duhr S, Braun D, Längst G, et al (2012) Microscale thermophoresis as a sensitive method to quantify protein: nucleic acid interactions in solution. Methods Mol Biol 815: 241–252. 22130996 10.1007/978-1-61779-424-7_18

[27] Coleman RA, Pugh BF (1995) Evidence for functional binding and stable sliding of the TATA binding protein on nonspecific DNA. J Biol Chem 270: 13850–13859. 7775443 10.1074/jbc.270.23.13850

[28] Maerkl SJ, Quake SR (2007) A Systems Approach to Measuring the Binding Energy Landscapes of Transcription Factors. Science 315: 233–237. 17218526 10.1126/science.1131007

[29] Baaske P, Wienken CJ, Reineck P, Duhr S, Braun D (2010) Optical Thermophoresis for Quantifying the Buffer Dependence of Aptamer Binding. Angew Chem Int Ed 49: 2238–2241. 10.1002/anie.200903998 20186894

[30] Becker PB, Wu C (1992) Cell-free system for assembly of transcriptionally repressed chromatin from Drosophila embryos. Molecular and Cellular Biology 12: 2241–2249. 1569951 10.1128/mcb.12.5.2241-2249.1992 PMC364396

[31] Langst G, Blank A, Becker PB, Grummt I (1997) RNA polymerase I transcription on nucleosomal templates: the transcription termination factor TTF-I induces chromatin remodeling and relieves transcriptional repression. The EMBO Journal 16: 760–768. 9049305 10.1093/emboj/16.4.760 PMC1169677

[32] Langst G, Becker PB, Grummt I (1998) TTF-I determines the chromatin architecture of the active rDNA promoter. The EMBO Journal 17: 3135–3145. 9606195 10.1093/emboj/17.11.3135 PMC1170652

[33] Shiue C-N, Berkson RG, Wright APH (2009) c-Myc induces changes in higher order rDNA structure on stimulation of quiescent cells. 28: 1833–1842. 10.1038/onc.2009.21 19270725

[34] Ling JQ, Li T, Hu JF, Vu TH, Chen HL, Qiu XW, Cherry AM, Hoffman AR (2006) CTCF Mediates Interchromosomal Colocalization Between Igf2/H19 and Wsb1/Nf1. Science 312: 269–272. 16614224 10.1126/science.1123191

[35] Stanojevic D, Small S, Levine M (1991) Regulation of a segmentation stripe by overlapping activators and repressors in the Drosophila embryo. Science 254: 1385–1387. 1683715 10.1126/science.1683715

[36] Arnone MI, Davidson EH (1997) The hardwiring of development: organization and function of genomic regulatory systems. Development 124: 1851–1864. 9169833 10.1242/dev.124.10.1851

[37] Papatsenko DA, Makeev VJ, Lifanov AP, Regnier M, Nazina AG, et al (2002) Extraction of Functional Binding Sites from Unique Regulatory Regions: The Drosophila Early Developmental Enhancers. Genome Research 12: 470–481. 11875036 10.1101/gr.212502 PMC155290

[38] Berman BP, Nibu Y, Pfeiffer BD, Tomancak P, Celniker SE, et al (2002) Exploiting transcription factor binding site clustering to identify cis-regulatory modules involved in pattern formation in the Drosophila genome. Proc Natl Acad Sci USA 99: 757–762. 11805330 10.1073/pnas.231608898 PMC117378

[39] Halfon MS, Grad Y, Church GM, Michelson AM (2002) Computation-based discovery of related transcriptional regulatory modules and motifs using an experimentally validated combinatorial model. Genome Research 12: 1019–1028. 12097338 10.1101/gr.228902 PMC186630

[40] Rye M, Sætrom P, Håndstad T, Drabløs F (2011) Clustered ChIP-Seq-defined transcription factor binding sites and histone modifications map distinct classes of regulatory elements. BMC Biology 9: 80. 22115494 10.1186/1741-7007-9-80 PMC3239327

[41] Vavouri T, Elgar G (2005) Prediction of cis-regulatory elements using binding site matrices — the successes, the failures and the reasons for both. Current Opinion in Genetics & Development 15: 395–402. 15950456 10.1016/j.gde.2005.05.002

[42] Somma MP, Pisano C, Lavia P (1991) The housekeeping promoter from the mouse CpG island HTF9 contains multiple protein-binding elements that are functionally redundant. Nucleic Acids Research 19: 2817–2824. 1711672 10.1093/nar/19.11.2817 PMC328238

[43] Giniger E, Ptashne M (1988) Cooperative DNA binding of the yeast transcriptional activator GAL4. Proc Natl Acad Sci USA 85: 382–386. 3124106 10.1073/pnas.85.2.382 PMC279552

[44] Lin YS, Carey M, Ptashne M, Green MR (1990) How different eukaryotic transcriptional activators can cooperate promiscuously. Nature 345: 359–361. 2188137 10.1038/345359a0

[45] Anderson GM, Freytag SO (1991) Synergistic activation of a human promoter in vivo by transcription factor Sp1. Molecular and Cellular Biology 11: 1935–1943. 2005889 10.1128/mcb.11.4.1935-1943.1991 PMC359878

[46] He X, Samee MAH, Blatti C, Sinha S (2010) Thermodynamics-Based Models of Transcriptional Regulation by Enhancers: The Roles of Synergistic Activation, Cooperative Binding and Short-Range Repression. PLoS Comput Biol 6: e1000935. 20862354 10.1371/journal.pcbi.1000935 PMC2940721

[47] Vicent GP, Zaurin R, Nacht AS, Font-Mateu J, Le Dily F, et al (2010) Nuclear Factor 1 Synergizes with Progesterone Receptor on the Mouse Mammary Tumor Virus Promoter Wrapped around a Histone H3/H4 Tetramer by Facilitating Access to the Central Hormone-responsive Elements. Journal of Biological Chemistry 285: 2622–2631. 19940123 10.1074/jbc.M109.060848 PMC2807319

[48] Adams CC, Workman JL (1995) Binding of disparate transcriptional activators to nucleosomal DNA is inherently cooperative. Molecular and Cellular Biology 15: 1405–1421. 7862134 10.1128/mcb.15.3.1405 PMC230365

[49] Kempers-Veenstra AE, Oliemans J, Offenberg H, Dekker AF, Piper PW, et al (1986) 3′-End formation of transcripts from the yeast rRNA operon. The EMBO Journal 5: 2703. 3780675 10.1002/j.1460-2075.1986.tb04554.x PMC1167172

[50] Cook PR (2003) Nongenic transcription, gene regulation and action at a distance. Journal of Cell Science 116: 4483–4491. 14576342 10.1242/jcs.00819

[51] Lykke-Andersen S, Mapendano CK, Heick Jensen T (2011) An ending is a new beginning: transcription termination supports re-initiation. Cell Cycle 10: 863–865. 21325895 10.4161/cc.10.6.14931

[52] Zentner GE, Saiakhova A, Manaenkov P, Adams MD, Scacheri PC (2011) Integrative genomic analysis of human ribosomal DNA. Nucleic Acids Res 39: 4949–4960. 21355038 10.1093/nar/gkq1326 PMC3130253

[53] Németh A, Strohner R, Grummt I, Längst G (2004) The chromatin remodeling complex NoRC and TTF-I cooperate in the regulation of the mammalian rRNA genes in vivo. Nucleic Acids Research 32: 4091–4099. 15292447 10.1093/nar/gkh732 PMC514363

[54] Seither P, Zatsepina O, Hoffmann M, et al (1997) Constitutive and strong association of PAF53 with RNA polymerase I. Chromosoma 106: 216–225. 9254723 10.1007/s004120050242

[55] Németh A, Conesa A, Santoyo-Lopez J, Medina I, Montaner D, et al (2010) Initial Genomics of the Human Nucleolus. PLoS Genet 6: e1000889 doi:10.1371/journal.pgen.1000889.g004 20361057 10.1371/journal.pgen.1000889 PMC2845662

[56] Langmead B, Trapnell C, Pop M, Salzberg SL (2009) Ultrafast and memory-efficient alignment of short DNA sequences to the human genome. Genome Biology 10: R25. 19261174 10.1186/gb-2009-10-3-r25 PMC2690996

[57] Heinz S, Benner C, Spann N, Bertolino E, Lin YC, et al (2010) Simple Combinations of Lineage-Determining Transcription Factors Prime cis-Regulatory Elements Required for Macrophage and B Cell Identities. Molecular Cell 38: 576–589. 20513432 10.1016/j.molcel.2010.05.004 PMC2898526

