# Supplementary figures and images for: Chromatin-Specific Regulation of Mammalian rDNA Transcription by Clustered TTF-I Binding Sites
Source: PLoS Genet. 2013 Sep 12;9(9):e1003786. doi: 10.1371/journal.pgen.1003786 (PMC3772059; doi:10.1371/journal.pgen.1003786)

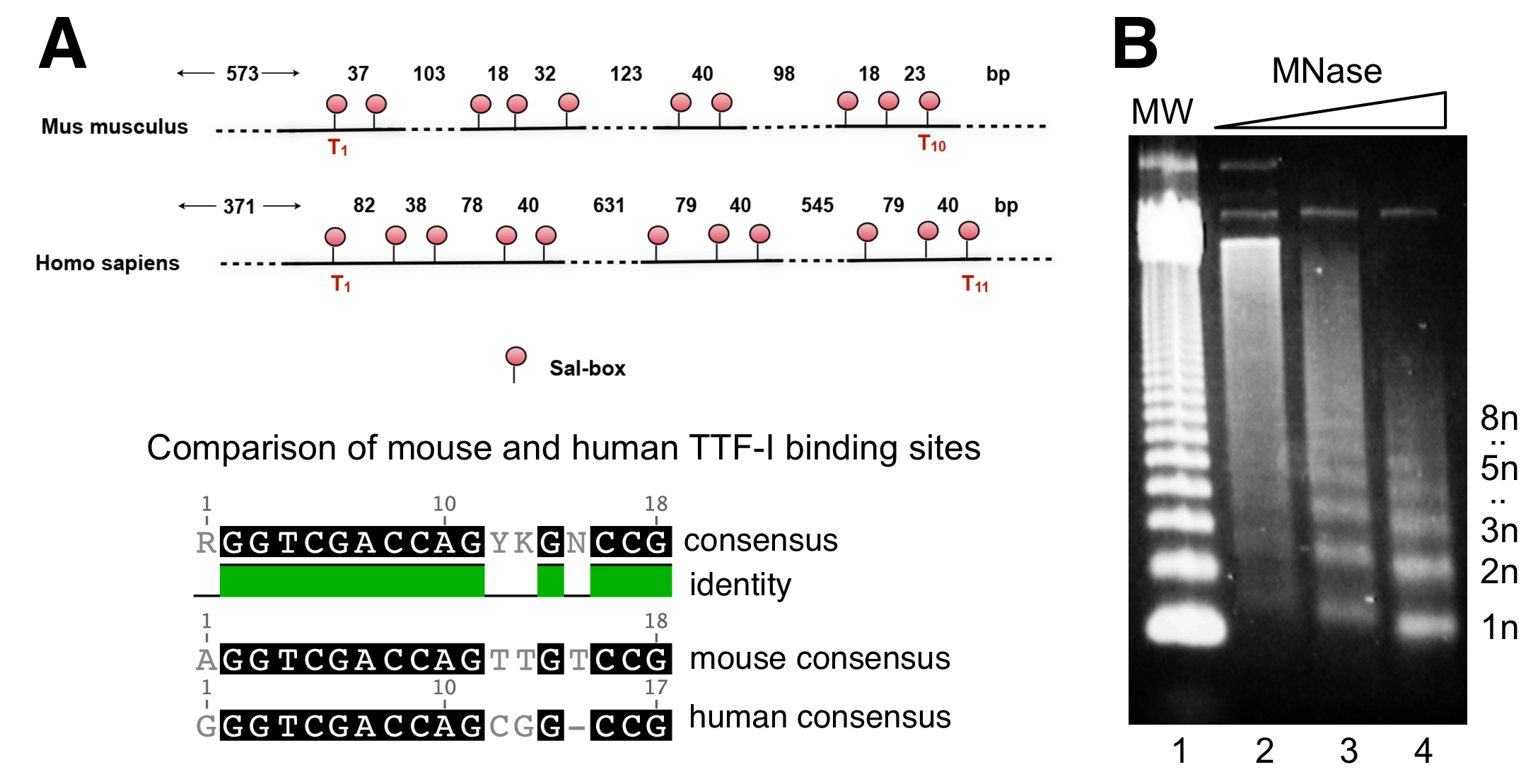

Supplement: Figure S1 — Related to Figure 1. Clustering of rRNA gene termination sites is evolutionary conserved. (A) Distribution of binding sites being involved in transcription termination of mouse and human rRNA genes. The relative distance to the end of the coding region and the distances between the individual binding sites are given. Lollipops mark TTF-I binding sites. Sequence comparison of the TTF-I binding sites in mouse and human is shown below. (B) MNase digestion of reconstituted chromatin. Chromatin was reconstituted with the Drosophila embryo extract and digested with increasing amounts of MNase. Purified DNA was visualized by agarose gel electrophoresis and ethidium bromide staining. The regular fragment ladder is indicative of an efficiently assembled nucleosomal array (1n through 8n). (JPG) [file pgen.1003786.s001.jpg]

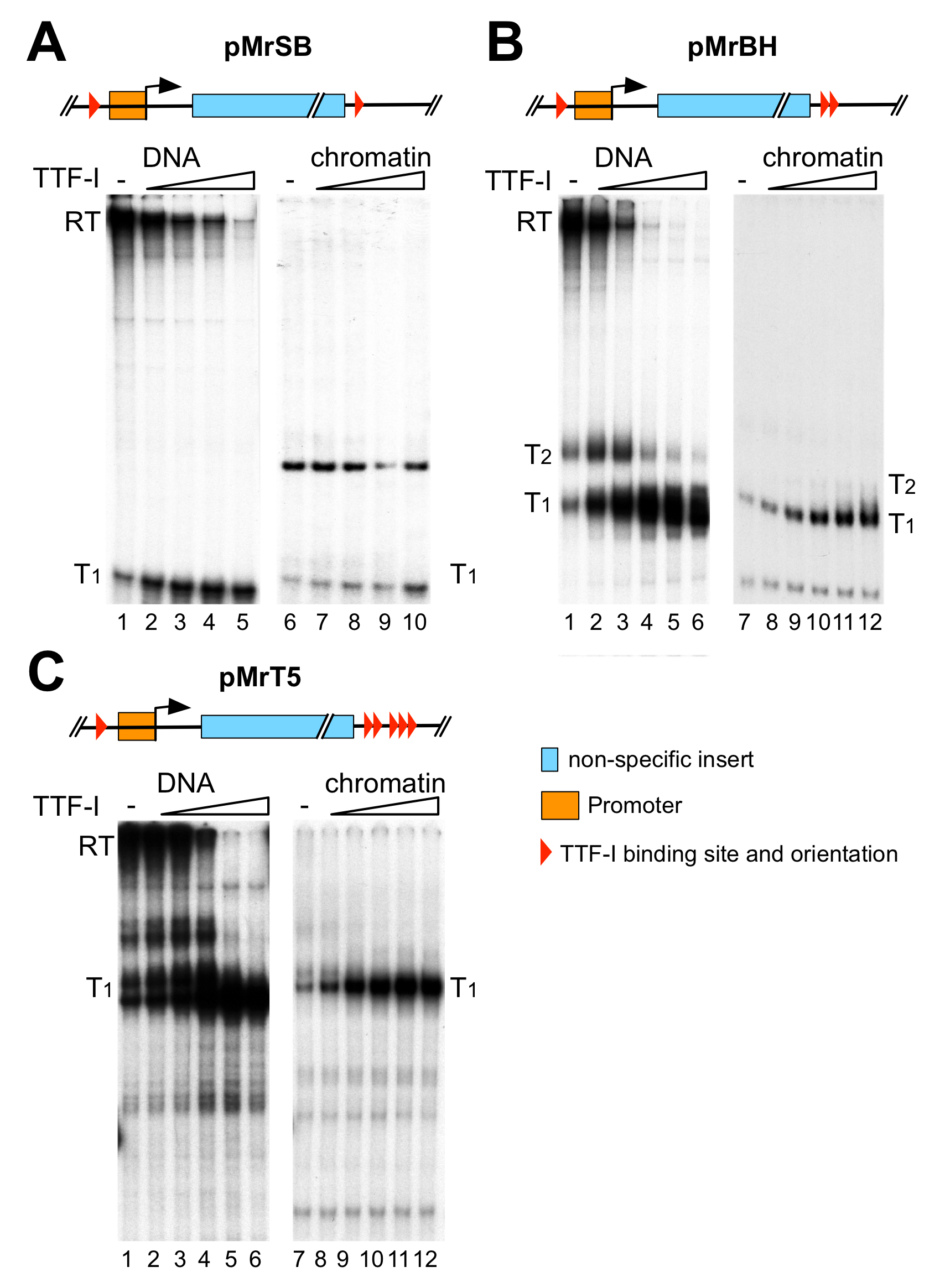

Supplement: Figure S2 — Related to Figure 1. Multiple termination sites are required for efficient transcription activation. (A) In vitro transcription analysis was performed comparatively on free DNA (lanes 1–5) or in vitro assembled chromatin (lanes 6–10) on pMrSB containing a single termination site (T1), either in the absence (lanes 1 and 6) or presence of TTF-I (lanes 2–5 and 7–10). The radioactively labeled transcripts were separated by PAA gel electrophoresis and detected by autoradiography. (B) In vitro transcription using the rRNA minigene pMrBH harboring the first two termination sites (T1+T2). The DNA was analysed for in vitro transcription on free DNA and chromatin with increasing amounts of TTF-I as described in (A). (C) In vitro transcription using the rRNA minigene pMrT5 harbouring the first five termination sites (T1 to T5). The DNA was analysed for in vitro transcription on free DNA and chromatin with increasing amounts of TTF-I as described in (A). (JPG) [file pgen.1003786.s002.jpg]

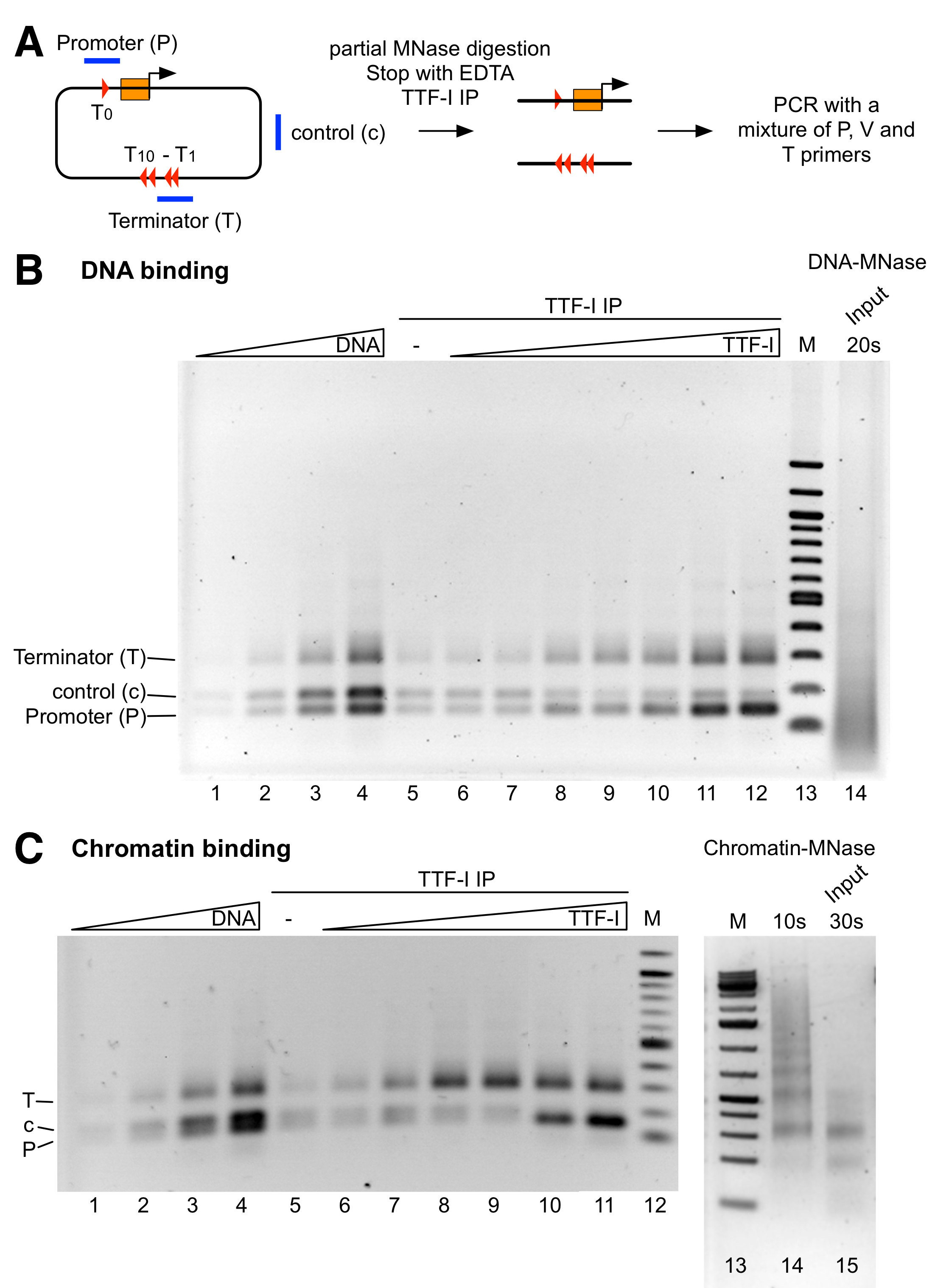

Supplement: Figure S3 — Related to Figure 2. TTF-I binds with higher affinity to the rDNA terminator in reconstituted chromatin. (A) Overview to the experimental strategy. The plasmid pMrEnLT10 containing the gene promoter, a 5 kb long transcribed region and the full terminator region was used for TTF-I binding experiments. Specific primers for PCR amplification of the regions containing T0 (Promoter, P, 145 bp), T1 to T3 (Terminator, T, 276 bp) and a control region of the vector (control, c, 187 bp) were designed. Primers were mixed to allow simultaneous detection and quantification of the three DNA regions. The plasmid was used as free DNA or reconstituted into chromatin with the Drosophila embryo extract. DNA or chromatin was incubated with TTF-I for 10 min and then partially digested with MNase (50 fmoles of DNA were incubated with 2 U MNase for 20 s; 300 ng of chromatin was incubated with 50 U MNase for 30 s; the reactions were stopped by the addition of EDTA to a final concentration of 5 mM). TTF-I bound DNA fragments were retained on Ni-NTA material in a batch assay and washed twice in Ex150 buffer. The associated DNA was purified and analysed by PCR using the mixture of primers. (B) Binding of TTF-I to the promoter and the terminator on free DNA. 50 fmoles of free DNA were incubated with increasing amounts of TTF-I (60 fmol to 4 pmol, lanes 6 to 12) and DNA was partially hydrolysed with MNase. A control digestion revealing the input DNA is shown in lane 14. Purified DNA was amplified with a mixture of primers giving rise to the Promoter (P), Terminator (T) and control (c) PCR fragments. Lanes 1 to 4 show the PCR amplification of increasing amounts of the partially digested pMrEnLT10 plasmid, revealing that the individual fragments were amplified with similar efficiency over a 16-fold concentration difference. Ni-NTA purification of the DNA in the absence of TTF-I gives rise to a background of PCR fragments (lane 5) that remains in the fractions containing increasing amounts of TTF- [file pgen.1003786.s003.jpg]

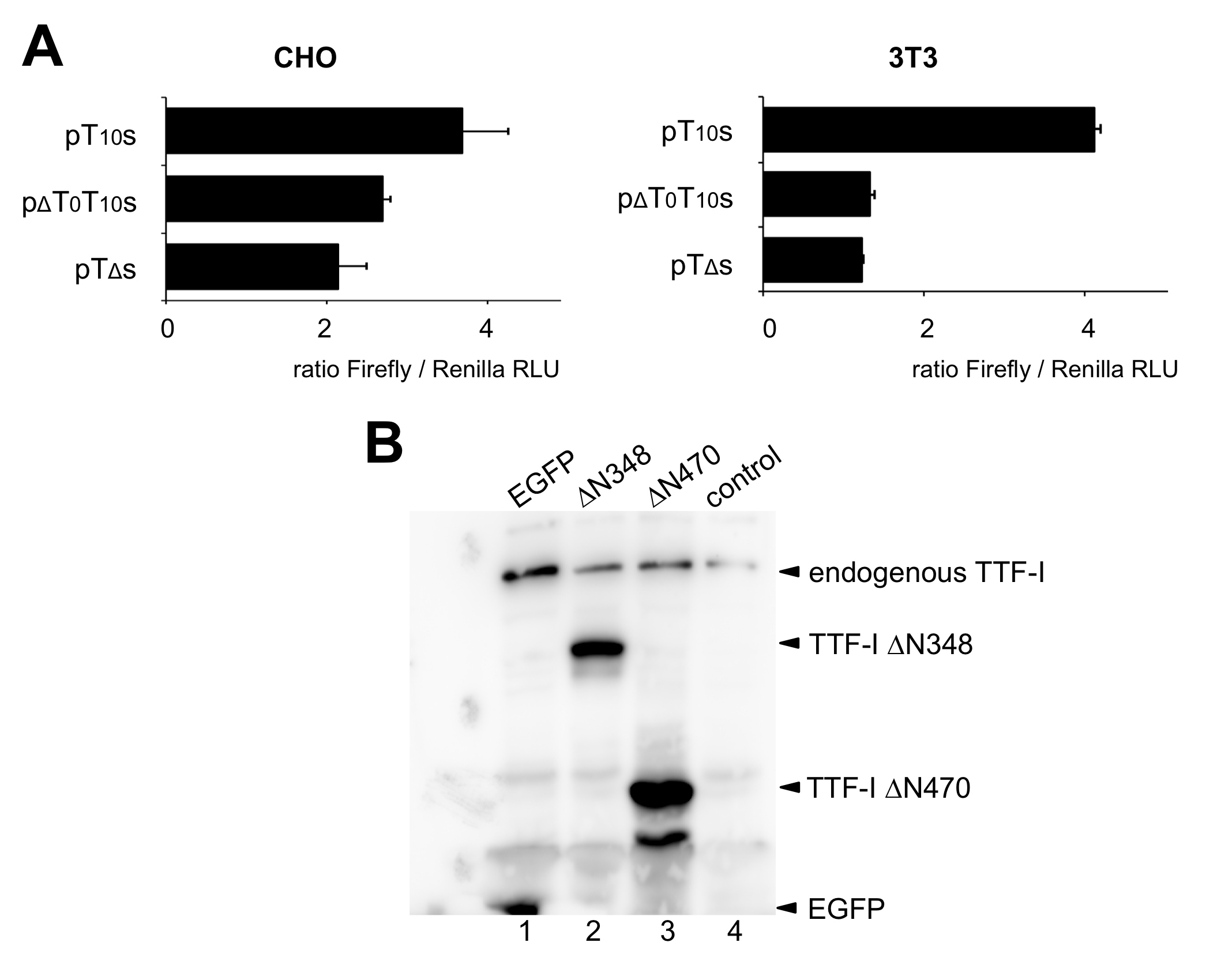

Supplement: Figure S4 — Related to Figure 3. Promoter-proximal and terminator TTF-I binding sites and the transactivation domain of TTF-I are required for full transcriptional activation of rRNA minigenes in vivo. (A) Transiently transfected rRNA minigenes contain mouse rDNA (BK000964) sequences from position −217 (pT10s, pTΔs) or −148 (pΔT0T10s) to +181, an IRES, the Firefly luciferase gene, and rDNA terminator regions from position +13169 to +15278 (pΔT0T10s and pT10s) in a pGL3-Basic vector (Promega). The plasmids contain a shorter non-specific insert than the constructs shown in Figure 3A. The insert size is 3 kilobases between the promoter and terminator region. CHO (left panel) or NIH3T3 cells (right panel) were transfected with Pol I driven Firefly luciferase reporter constructs and a Pol II Renilla luciferase control plasmid, pRL-TK (Promega). Reporter gene measurements were performed using the Dual Luciferase Reporter Assay System (Promega). Deletion of either the promoter-proximal or the terminator TTF-I binding sites reduces transcriptional activity, complementing the results shown in Figure 3. (B) Western Blot of transiently transfected CHO cells expressing EGFP-tagged TTF-I deletion mutants used in Figure 3B and C. Detection was performed with an α-GFP (sc-8334) and subsequently an α-TTF-I antibody (αC7). Lane 2: control transfection with a vector expressing only EGFP, lanes 3–4: overexpressed EGFP-tagged TTF-I ΔN348 or TTF-I ΔN470, lane 5: non-transfected control CHO cells. Endogenous full-length TTF-I is visible in all lanes. MW = molecular weight marker. (JPG) [file pgen.1003786.s004.jpg]

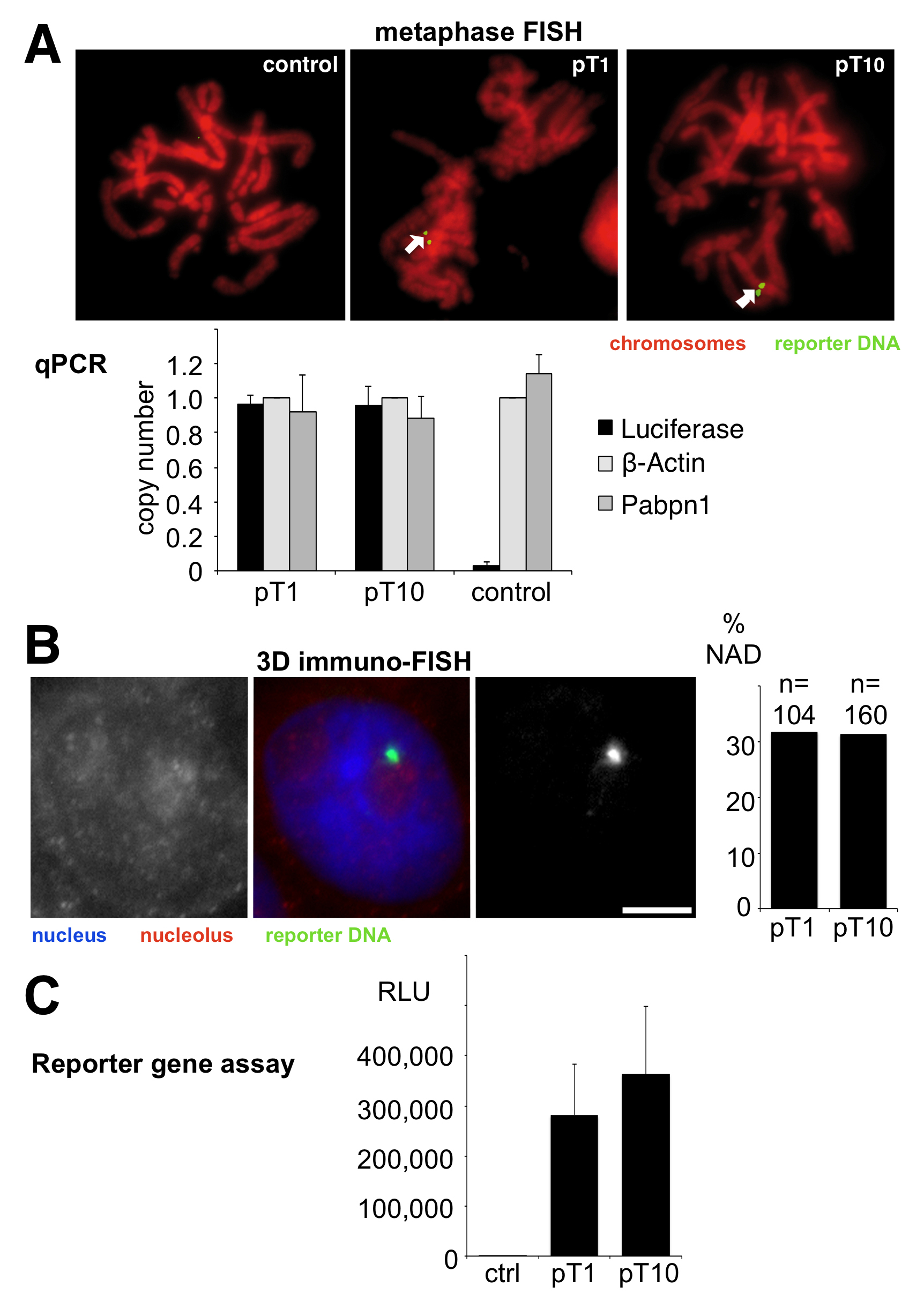

Supplement: Figure S5 — Related to Figure 4. Characterization of stable cell lines containing a single mouse rRNA gene. rRNA minigenes containing one or ten termination sites (pT1 and pT10) were genomically inserted into CHO Flp-In cells and stable single integrants were selected. This resulted in the cell lines CHO-pT1 and CHO-pT10. In all experiments, non-transfected CHO Flp-In cell lines were used as controls. Bars represent the mean of three independent stable transfections and error bars indicate standard deviations. (A) FISH detection of genomically inserted mouse rRNA minigenes on CHO Flp-In metaphase spreads. Chromosomes were stained with DAPI and are illustrated in red. Hybridization signals of reporter probes are shown in green. Arrows indicate the single genomic insertion site. The lower panel shows copy number determination of the integrated rDNA reporter plasmids. qPCR was performed on genomic DNA and comparative quantitation was performed between the luciferase gene and the copy number of two single-copy housekeeping genes, β-actin and PabpnI. Bars represent the mean of two independent experiments, error bars denote standard deviations. (B) The number of termination sites does not influence localization of the rDNA minigenes. 3D immuno-FISH analysis of genomically inserted pT1 and pT10 in interphase nuclei. Nuclear DNA was stained with DAPI (shown in blue in the middle merged panel), nucleoli with an α-B23 antibody and indirect immunofluorescence (left panel, and shown in red in the middle merged panel), and the rRNA minigenes were visualized by FISH (right panel, and shown in green in the middle merged panel). Bars depict the percentage of genomically integrated minigenes associated to the nucleolus, n denotes the absolute number of assayed alleles. Bar: 5 µm. (C) Firefly luciferase reporter gene assay on genomically integrated rRNA minigenes. Relative light units (RLU) were measured in three independent experiments, error bars indicate standard deviations. As control, non-t [file pgen.1003786.s005.jpg]

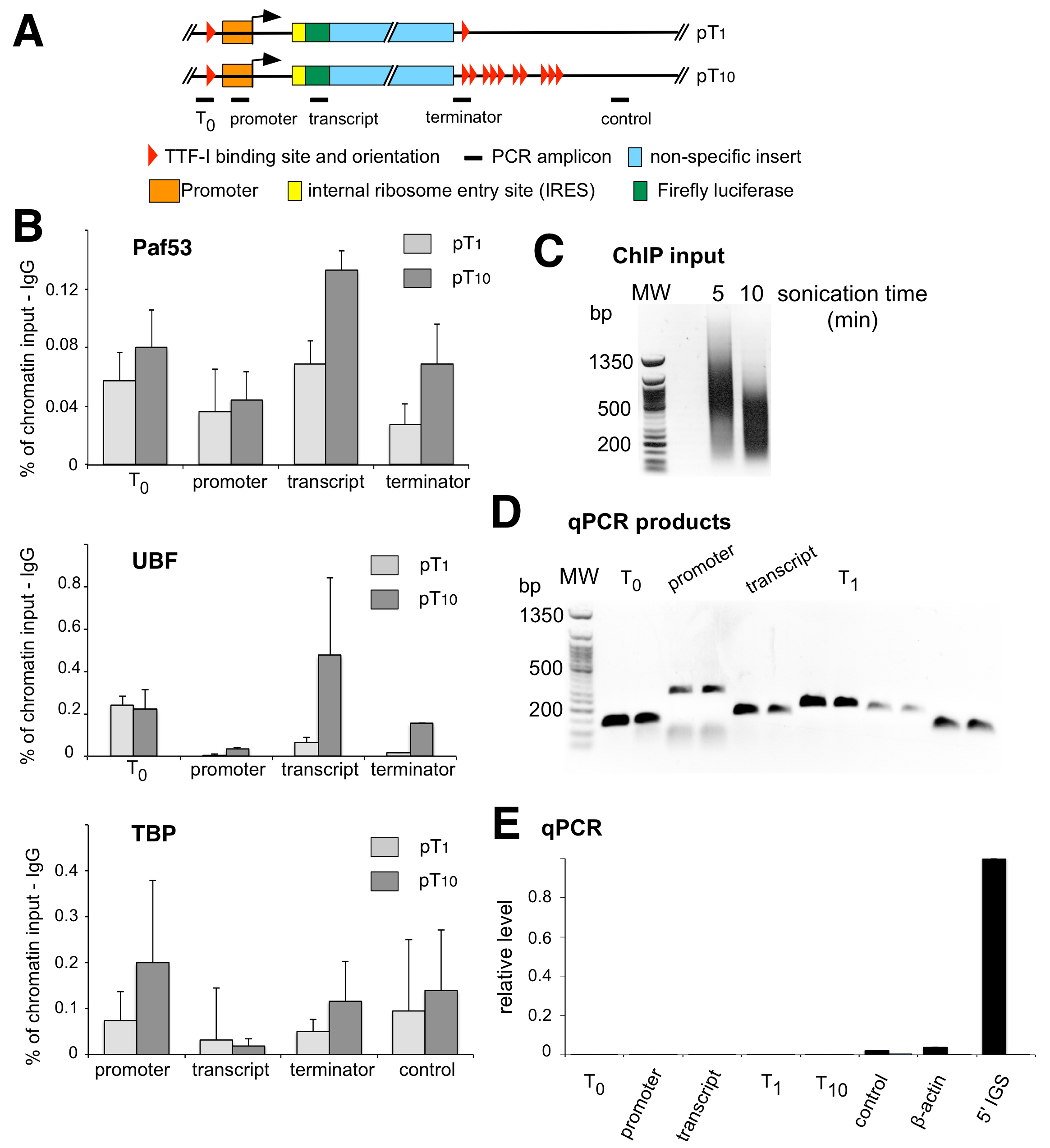

Supplement: Figure S6 — Related to Figure 4. ChIP experiments in transiently transfected CHO cells. (A) Overview of rDNA minigenes and the locations of the PCR amplicons. (B) Chromatin-immunoprecipitation (ChIP) assays on transiently transfected rDNA reporter genes using the indicated antibodies. Occupancies were measured by qPCR, calculated as percentage of input chromatin and background signals as determined from control IPs with unspecific antibodies (α-IgG or α-HA Tag) were subtracted. Three independent biological replicates were performed. Error bars indicate the standard error of the mean. (C) Sonication test. Representative agarose gel of the chromatin input sonicated for 5 or 10 min (30 sec on/30 sec off, settings: “high”) after proteinase K digestion and reversal of crosslinking. 10 min sonication time was used for all experiments. Fragment size range: 100–600 bp. MW = molecular weight marker. (D) Representative agarose gel of qPCR amplicons, pipetted in duplicates, after 40 cycles of qPCR. MW = molecular weight marker. (E) Mouse-specific primer pairs were tested on non-transfected CHO cells to ensure species-specific amplicons. Chromatin was isolated from CHO cells, processed like an input for ChIP experiments and analysed by qPCR. DNA levels were normalised to the 5′ IGS signal of hamster rDNA (5′ IGS). The multi-copy rRNA genes show a 25-fold higher signal than the single-copy gene β-actin. None of the mouse specific primer pairs amplifies detectable products on hamster chromatin. A faint signal appears in the plasmid-specific control primer pair. Each bar represents the mean of three replicates. For every primer pair, both CHO chromatin template triplicates (left) and water control (right) are shown. (JPG) [file pgen.1003786.s006.jpg]

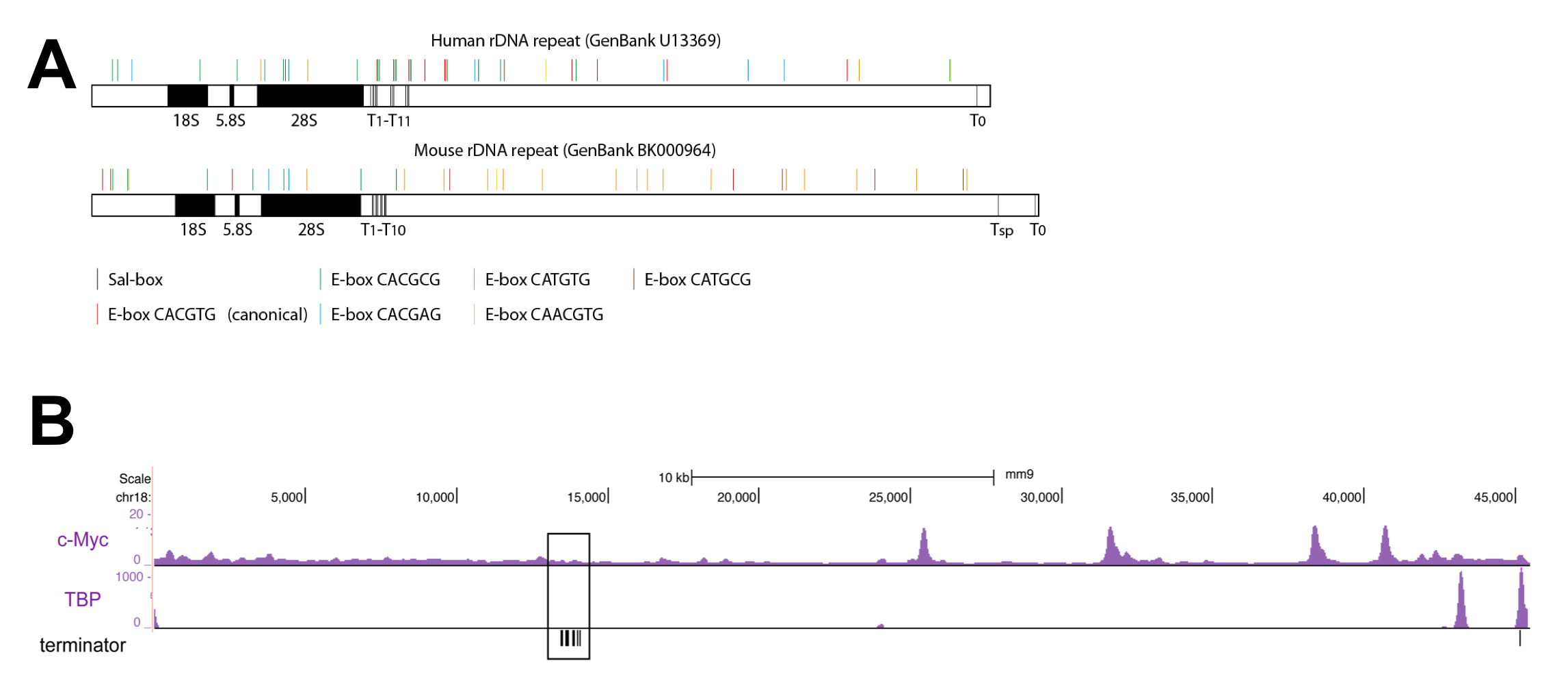

Supplement: Figure S7 — Related to Figure 5. Distribution E-boxes, c-Myc and TBP at the murine rDNA. (A) In silico comparison of the human and mouse rDNA repeat. The murine terminator region comprising of T1 to T10 does not overlap with E-box elements, the canonical c-Myc binding sites. (B) Enrichment of histone modifications at rDNA in MEL cells. The whole rDNA repeat is plotted from position +1 (the TSS) to position 45.500. The terminator track indicates TTF-I binding sites by black vertical lines. The black box highlights the clustered terminator elements at the 3′ end of the gene. ChIP-Seq tracks of c-Myc and TBP display relative enrichments compared to input. (JPG) [file pgen.1003786.s007.jpg]
