# Supplementary material for: Chromatin-Specific Regulation of Mammalian rDNA Transcription by Clustered TTF-I Binding Sites
Source: PLoS Genet. 2013 Sep 12;9(9):e1003786. doi: 10.1371/journal.pgen.1003786 (PMC3772059; doi:10.1371/journal.pgen.1003786)
Supplement: Table S1 — Summary of published NGS data used in this study. The table provides an overview of all next-generation sequencing datasets that have been used in the study. Cell types, accession numbers and respective publications are indicated for each dataset. The number of reads indicates absolute tag counts of sequencing reads mapped to the expanded reference genome. (DOC) [file pgen.1003786.s008.doc]

**Table S1**

Summary of published NGS data used in this study

| **Cell Type** | **GEO acc.** | **Publication** | **ChIP-Seq**  **(Epitope)** | **Total mapped tags (mm9_rDNA)** |
| --- | --- | --- | --- | --- |
| 3T3-L1 | GSM535745 | PMID:20887899 | H3K27me3 | 13,404,633 |
|  | GSM535744 | PMID:20887899 | H3K27ac | 12,674,683 |
|  | GSM535743 | PMID:20887899 | H3K4me1 | 12,342,203 |
|  | GSM535742 | PMID:20887899 | H3K4me2 | 11,931,787 |
|  | GSM535741 | PMID:20887899 | H3K4me3 | 8,889,170 |
|  | GSM535740 | PMID:20887899 | Input | 18,758,197 |
| MEL | GSM912913 | Mouse ENCODE | Tbp | 43,612,615 |
|  | GSM912934 | Mouse ENCODE | cMyc | 41,319,733 |
|  | GSM912916 | Mouse ENCODE | Input | 57,159,677 |
