# Supplementary material for: Chromatin-Specific Regulation of Mammalian rDNA Transcription by Clustered TTF-I Binding Sites
Source: PLoS Genet. 2013 Sep 12;9(9):e1003786. doi: 10.1371/journal.pgen.1003786 (PMC3772059; doi:10.1371/journal.pgen.1003786)
Supplement: Table S2 — List of qPCR primers used for ChIP analyses. The primer lists contains all primers used for quantitation of ChIP assays. Name, binding site, sequence and annealing temperatures are provided for each primer pair used in the study. (DOCX) [file pgen.1003786.s009.docx]

**Table S2**

List of qPCR primers used for ChIP analyses

| **Name** | **Binding site** | **Sequence** | **Tm** |
| --- | --- | --- | --- |
| LucTx_F | transcribed region of rRNA minigene | CAAGATTCAAAGTGCGCTGCTGGT | 62 |
| LucTx_R | transcribed region of rRNA minigene | TTGCCTGATACCTGGCAGATGGAA | 62 |
| LucPI_F | plasmid backbone of rRNA minigenes (control) | ACTATCGTCGCCGCACTTATGACT | 62 |
| LucPI_R | plasmid backbone of rRNA minigenes (control) | ACCGTATTACCGCCTTTGAGTGAG | 62 |
| Hygro_F | hygromycin CDS (control Flp-In) | GAGCGAGGCGATGTTCGGGG | 62 |
| Hygro_R | hygromycin CDS (control Flp-In) | TCCGGATGCCTCCGCTCGAA | 62 |
| MrT_1__F | T_1_ of Mm rDNA | AGGCTGTTGGTGAAACTGCT | 58 |
| MrT_1__R | T_1_ of Mm rDNA | AAGGAGCTGACTGGGTTGAA | 58 |
| MrT_10__F | T_6-7_ of Mm rDNA | CCTTTACTCTTCCCCACAGCGATTC | 60 |
| MrT_10__R | T_6-7_ of Mm rDNA | CGGGACACTTTCGGACATCTGG | 60 |
| MrT_0__F | T_0_ of Mm rDNA | GGAAAGCTATGGGCGCGGTT | 58 |
| MrT_0__R | T_0_ of Mm rDNA | AAGTCATACCTGGGGAGGTGGC | 58 |
| MrP'_F | gene promoter of Mm rDNA | CGACCAGTTGTTCCTTTGAGGT | 60 |
| MrP'_R | gene promoter of Mm rDNA | ACAGCTTCAGGCACCGCGAC | 60 |
| TATA_F | Pol II promoter (TATA-box) | AGCGGTTTGACTCACGGGGA | 60 |
| TATA_R | Pol II promoter (TATA-box) | CACCGTACACGCCTACCGCC | 60 |
